# Exploring the efficacy of *Cystoseira sedoide* alga for cadmium and copper biosorption: an integrated experimental and computational study†

**DOI:** 10.1039/d4ra07331b

**Published:** 2024-12-10

**Authors:** Nadjette Bengourna, Karima Belguidoum, Dounya Khalla, Mouna Nacef, Imane Kouadri, Aida Benhamida, Habiba Amira-Guebailia, Alaa eddine Brouk, Abed Mohamed Affoune, Hamid Satha

**Affiliations:** a Laboratoire des Silicates, Polymères et des Nanocomposites (LSPN), Université 8 Mai 1945, Guelma BP 401 Guelma 24000 Algeria; b Laboratoire de Chimie Appliquée (L.C.A), Université 8 Mai 1945 BP 401 24000 Guelma Algeria; c Laboratoire d'Analyses Industrielles et Génie des Matériaux (L.A.I.G.M), Université 8 Mai 1945 Guelma BP 401 Guelma 24000 Algeria nacef.mouna@univ-guelma.dz nacef2010@yahoo.fr

## Abstract

Pollution by heavy metals is a major global issue. The biosorptive removal of Cd^2+^ and Cu^2+^ by *Cystoseira sedoide* (*C. sedoide*) was evaluated in this work. FTIR and XRD analysis were performed to determine the characteristics of the biosorbent. In batch biosorption studies, several operating parameters such as solution pH and concentration, contact time, biosorbent dose and temperature were tested and optimized for the effective elimination of cadmium and copper ions. Results were further compared to industrial activated carbon (Ac.C). The biosorption capacities for Cd^2+^ and Cu^2+^ were 23.78 and 14.66 mg g^−1^, respectively. Excellent removal rates were achieved for both Cd^2+^ and Cu^2+^ by *C. sedoide*. In experiments with varying temperature, biosorbent dose, and heavy metal ions concentration, almost steady states were observed whatever the operating conditions and no notable differences were observed within the studied range of conditions. However, Ac.C performance was dependent on the operating conditions. Moreover, cadmium ion removal by *C. sedoide* was efficient even in the presence of copper ions. Based on the density functional theory (DFT) computations, it can be stated that the attraction forces between the heavy metal ion and the biosorbent depend on the considered structure and arrangement of the proposed complex models. Besides the revealed benefits of using *C. sedoide* for the removal of heavy metals, the biomass can be reused as a feedstock for the production of biochar or bioethanol, leading to economic and environmental sustainability when implemented on a large scale.

## Abstract 2

Heavy metals biosorption by *Cystoseira sedoide* alga.

## Graphical abstract

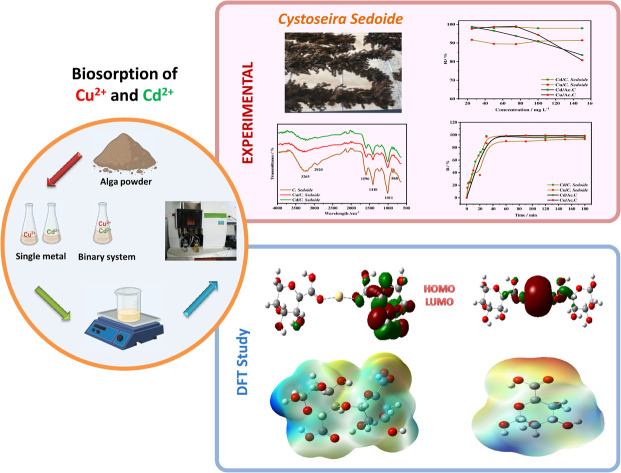

## 1. Introduction

Polluted water sources with heavy metals are harmful to aquatic life. Humans are doubly impacted by water pollution; directly by the use of contaminated water or indirectly by fish and other sea product consumption.^1^ In fact, the harmful accumulative behaviour of such metals in aquatic organisms has been raised by the scientific community over the past decades.^2,3^

Among thousands of industrial chemical compounds, heavy metals cause the death and disappearance of many species, weakening thus the ecosystem. Although heavy metals occur naturally in the environment such as from rainfall, dissolution of soluble salts and bedrock erosion, their release from anthropogenic activities is one of the key environmental problems facing humanity. Indeed, the aquatic compartment receives large amounts of these compounds from wastewater, agriculture sector, and industrial activities.

Even though some metals are essential for humans' metabolism, the continuous exposure to high levels of heavy metals is perilous to humans and to other living species. Heavy metals are known as having high density, to be non-biodegradable and toxic at very low concentrations. Copper has a key role in human body growth, development and reproduction. But if human body is subjected to higher levels of this substance, several disorders may arise such as brain and kidney damage, gastrointestinal issues and even liver cirrhosis or chronic anemia.^4^ On the other hand, cadmium is carcinogenic and could induce kidney damage.^5^ It is mutagenic, endocrine disruptor inducing lung damage and weak bones. It has acute effects in children and affects calcium regulation in biological systems.^6^ Copper can be found near copper mining, steel, etching, and electroplating industries,^7^ while cadmium is mainly present in surface waters by transfer from various chemical industries.^8^ Facing this situation, Food and Agriculture Organization of the United Nations (FAO) guidelines stipulate that copper and cadmium levels in irrigation water must not exceed 10 and 200 μg L^−1^, respectively.^9^ While the World Health Organization's recommendations for these two metals limit their levels in drinking water to 1.5 mg L^−1^ and 0.003 mg L^−1^ for Cu^2+^ and Cd^2+^, respectively.^10^

Water treatments from heavy metals contamination have been conducted through many routes like chemical precipitation, coagulation floculation, ion exchange, membrane filtration, and electrochemical processing. All these techniques have advantages and disadvantages but the most important disadvantage is their cost. So, adsorption appears cheaper and easier to install and implement.^11^ Among several adsorbents, activated carbon is the most used due to its recognized availability and efficiency.^12^ So a tremendous number of papers are dedicated every year to the adsorption phenomenon; Fig. S1,† where adsorbents are mainly agriculture raw or modified materials which are often selected from the surrounding environment, making the activated carbon elaboration cost-effective.^13–15^

The use of algae in heavy metals uptake was considered only since the three past decades as a readily available and renewable resource Fig. S2.† The Freshwater Algal Flora of the British Isles recognizes 15 phyla and some of them were investigated as potential adsorbents.^16,17^ The *Phaeophyta phylum*, encompasses brown algae such as *Laminariaceae*, *Fucaceae*, *Sargassaceae*, *Dictyotaceae*, and others.^18^ The use of brown algae species as biosorbent was reported early in the 2000s.^19^ For instance, some metal ions removal was investigated using brown algae such as; *Turbinaria ornata*,^20^*Fucus spiralis*, *Fucus versiculosus*, and *Polysiphonia lanosa*^21^ which were assessed for copper removal. *Fucus spiralis*, *Fucus versiculosus*, and *Polysiphonia lanosa*^22^ were successfully employed for chromium elimination and *Sargassum natans*, *Fucus versiculosus*, *Ascophyllum nodosum*,^23^ and *Fucus vesiculosus*^24^ were used in cadmium biosorption.

*Cystoseira sedoide* is an endemic species of the Mediterranean sea, occurring mainly in the Algerian and Tunisian coastline. It can be found at low depths not deeper than 1.5 meters with a significant settlement.^25^ Therefore, obtaining large amounts of *Cystoseira sedoide* is simple. It was reported that *C. sedoide*, as well as marine brown algae are constituted of alginate as the major compound^26^ with carbon and oxygen elements having a percentage of around 89% but also many other minerals such as K, Ca, Si, S, Fe, Na, Mg, P, as well as some heavy metals (Cu, Ni, Zn).^27^ Usually, alginate contains high molecular weight linear polysaccharides where two monosaccharides units (uronic acids) β-d-mannuronic acid (M) and α-l-guluronic acid (G) which are linked to each other by (1 → 4) glycosidic bonds to form a linear chain comprising G, M, MG, and GM blocks. These two units have a *quasi*-similar structure and differ only by the position of the carbonyl function. Guluronic acid is an epimer of mannuronic acid at the C-5 position. The functional groups on the alginate surface have sometimes a negative charge which is balanced by the presence of ions like Ca^2+^, Na^+^, Mg^2+^, *etc.* Heavy metals removal by algae could occur by an ion exchange with these ions^28^ or as recently stated by the formation of complexes between algae and heavy metals.^29,30^

Since the past few years, and thanks to the rapid growth of various numerical simulation systems, the theoretical approach has been considered as a valuable tool for both the prediction and the elucidation of the mechanism by which the pollutant was linked to the adsorbent.^31,32^ In this context, the interaction between the targeted heavy metals and the adsorbent was studied by optimizing and considering a representative functionalized model of the adsorbent.^33,34^

For the first time, the use of an air dried and without further treatment *Cystoseira sedoide* as biosorbent for cadmium and copper ions elimination from aqueous solutions is reported herein. To better evaluate the algal biosorption performances, this former was compared to industrial activated carbon in Cd^2+^ and Cu^2+^ adsorption. Furthermore, a theoretical investigation using DFT computations was carried out with the assumption of the formation of a complex between two different alginate configurations and cadmium and copper ions.

## 2. Materials et methods

### 2.1. Materials and reagents

Brown *Cystoseira sedoide* alga was collected in the Algerian coastal (Tizi Ouzou region) in December. Commercial spherical activated carbon grade A-BAC MP from KUREHA (Tokyo, Japan) was kindly provided by SONATRACH. Copper(ii) sulfate pentahydrate (CuSO_4_·5H_2_O) and sodium hydroxide (NaOH) were bought from Sigma-Aldrich, cadmium nitrate tetrahydrate Cd(NO_3_)_2_·4H_2_O was purchased from BIOCHEM, nitric acid (HNO_3_) and hydrochloric acid (HCl) were supplied by MERCK. Stock solutions of 500.0 mg L^−1^ were prepared of both Cd^2+^ and Cu^2+^ and stored without any pH adjustment. The test solutions were prepared by appropriate dilution of the stock solutions. In the section related to the pH influence on the adsorptive removal of heavy metals ions, pH was adjusted using HCl (1 M) or NaOH (1 M).

The brown alga was thoroughly washed with sea water to remove sand and any other impurities and then with deionised water. After that, alga was left to dry at room temperature in the shade for 15 days then grinded with an electric grinder and sieved to ensure that the *C. sedoide* powder was at approximately at the same size (0.4–0.5 mm). Finally, *C. sedoide* powder was stocked in a PEHD sealed container until use.

### 2.2. Apparatus and *C. sedoide* characterization

XRD patterns were obtained using a D2 phaser X-ray diffractometer. Bruker AXS, Karlsruhe, Germany. Alga samples were examined with the 2*θ* range from 10° to 55° at an increment of 0.01°. The functional groups of *C. sedoide* alga, before and after being mixed with heavy metal ions solutions, were analyzed using FT-IR spectra acquired by AAnalyst 1600 FTIR spectrophotometer. PerkinElmer, Waltham, MA, USA. The scan range was varied from 4000 cm^−1^ to 500 cm^−1^ with a resolution of 4 cm^−1^ using ten replicates. Metal ions concentrations after the adsorption process were measured using an AAnalyst 4000 atomic absorption spectrometer. PerkinElmer, Waltham, MA, USA. Biosorbent/metal ions solution separation was conducted using a centrifuge apparatus Sigma 2–7 centrifuge (rotor size: 4 × 400 mL). Sartorius AG, Göttingen.

### 2.3. Adsorption assays

The effect of contact time, solution pH, temperature, initial concentration and the biosorbent dose on Cd^2+^ or Cu^2+^ elimination in aqueous solution was investigated as they play a crucial role in their biosorption. A weighted amount of *C. sedoide* powder was dispersed in the aqueous solutions containing either Cd^2+^ or Cu^2+^ ions or both of them under stirring at a constant speed of 300 rpm and a chosen temperature until reaching the equilibrium state. Stirring solution is of outmost importance since the prior results revealed that the biosorption raised of about 5 to 12% after agitation; Fig. S3.† Afterward, the mixture was separated using centrifugation at 4000 rpm. The supernatant was then adequately diluted and subjected to the atomic absorption spectroscopy in order to determine cadmium and copper ions concentration before and after biosorption. In order to assess *Cystoseira sedoide* efficiency for an actual application, the adsorption experiments were also carried out using commercial activated carbon in order to make an adequate comparison. To this end, the same procedure was followed when industrial activated carbon was used as adsorbent.

To determine the optimal biosorbent dose value for both metal ions biosorption, 0.2, 0.4, 0.5, 0.8, 1.0, 2.0, and 3.0 g of *C. sedoide* powder were mixed with 25 mL of Cd^2+^ and Cu^2+^ solutions (50 mg L^−1^). Temperature was set at 25, 30, 40, 50 and 60 °C by keeping the biosorbent dose/solution volume ratio as 0.5g/25 mL. Similarly, Cd^2+^ and Cu^2+^ kinetics curves were constructed with an initial metal ion concentration of 50 mg L^−1^ and the contact time between *C. sedoide* powder and the metallic solution was increased from 2 min to 180 min. The isotherm fitting curves were obtained by varying the initial metal ions concentration (25–150 mg L^−1^). For both kinetics and thermodynamic assays, the pH and adsorbent dose were kept unchanged (*m*: 0.5 g, *V*: 25 mL, *C*_0_: 50 mg L, *t*: 180 min).

The adsorption capacity (*q*_e_ (mg g^−1^)) and the removal rate (*R*%) of Cd^2+^ and Cu^2+^ onto *C. sedoide* powder, as well as onto Ac.C were calculated using the following equations:^35,36^1
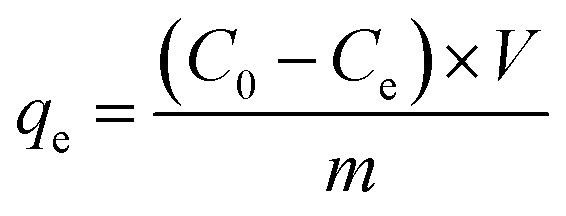
2
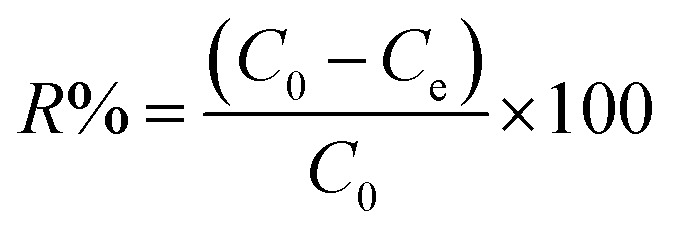
The parameters *C*_0_ and *C*_e_ denote, respectively, the initial and equilibrium concentration (mg L) of the Cd^2+^ and Cu^2+^ solution. *V* and *m* represent the volume of the heavy metal ions solution (L) and the *C. sedoide*/activated carbon mass (g), respectively.

### 2.4. Cadmium and copper ions co-biosorption experiments

For the purposes of investigating the effect of copper ions occurrence in the same solution containing Cd^2+^ on the adsorptive removal of this latter, as it's by far more harmful than copper ions, both metals biosorption was carried out using an aqueous solution with concentrations: (Cd^2+^) = (Cu^2+^) = 50 mg L^−1^. The other experiment conditions were: *m*: 1.0 g, *V*: 50 mL, and *t*: 120 min.

### 2.5. Desorption assays

After an adsorption experiment for a duration of 180 min in the following conditions: *V* = 25 mL, *C* = 50 mg L^−1^, *m* = 0.5 g, stirring speed = 300 rpm, and alga filtration and drying, a desorption assay was conducted on a portion of the dried alga using HNO_3_ (1%) with the following conditions: *m*/*V* = 0.05 g/25 mL, contact time 180 min and without stirring. After filtration, the solution concentration was measured by AAS to determine the recovered heavy metal ions percentage.

### 2.6. DFT analysis

Beside the experimental study, DFT approach was employed to study the affinity of *C. sedoide* for cadmium and copper ions. Gaussian 09 program package coupled with Gaussview was used to perform the DFT computations. The optimization of the different structures was performed at B3LYP-D3/LanL2DZ G(d,p) level of theory which is considered as a suitable and reliable level for the prediction of the complexes properties.^37^

As stated above, and according to the bulk alginate structure, two potential routes for cadmium and copper biosorption to the functional groups were considered. First, cadmium or copper ion was placed between two monosaccharides (M). For each heavy metal, two conformations were examined; the symmetric and the inverse one. The second configuration was as follows: two linked (M) units were considered as the smallest biopolymer block (dimer) that could react with the two heavy metal ions. In this case, two heavy metal ions were linked to the adjacent side of the dimer as follows: (Cu-ALG-Cu)^4+^, (Cd-ALG-Cd)^4+^, (Cu-ALG-Cd)^4+^. The latter configuration allows the modelling of the heavy metals co-adsorption studied experimentally. The studies were conducted in the aqueous state by considering the protonated form of the monosaccharides, as well as for the dimer.

Based on the FTIR results, the carbonyl groups of the uronic acids were more likely prone to be linked with heavy metal ions. Before the simulation calculations, the functionalized (M) unit and MM dimer were optimized. Then, organometallic complexes were generated between the alginate configuration model and heavy metals. The theoretical study was performed by the determination of HOMO (Energy of the Highest Occupied Molecular Orbital) and LUMO (Energy of the Lowest Unoccupied Molecular Orbital) boundary orbital's energies and molecular electrostatic potentials (MEP). Also, the adsorption energies were calculated using the following expressions:

3 Δ*E* = *E*_complex_ − (2 × *E*_ligand_) − *E*_metal_

Eqn (3) was used for the complexes Cu(ALG)^2+^/symmetry, Cu(ALG)^2+^/inverse, Cd(ALG)^2+^/symmetry, Cd(ALG)^2+^/inverse.

For complexes (Cu-ALG-Cu)^4+^ and (Cd-ALG-Cd)^4+^, the following eqn (4) was employed:

4 Δ*E* = *E*_complex_ − *E*_ligand_ − (2 × *E*_metal_)

The eqn (5) is applicable for the (Cu-ALG-Cd)^4+^ complex:

5 Δ*E* = *E*_complex_ − *E*_ligand_ − (*E*_metal(Cu)_ + *E*_metal(Cd)_)

where; *E*_complex_ is the total energy for the complex formed after adsorption, *E*_ligand_ is the adsorbent energy, and *E*_metal_ is the energy of the cadmium or copper ion.

Chemical interactions were described using various parameters. They are listed below:^38^6
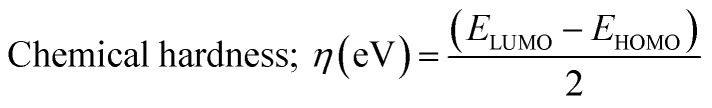
7
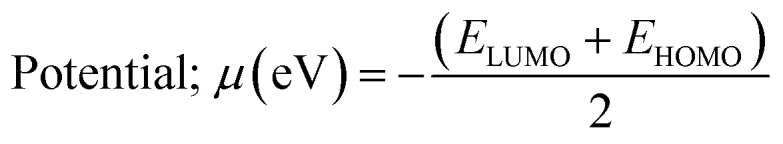
8
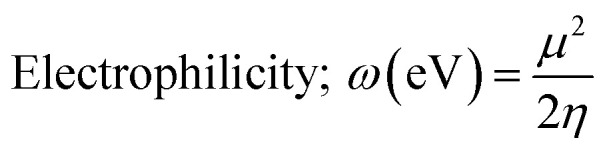


The IBSI value (Intrinsic Bond Strength Index) is a parameter used to quantify the bond strength in molecular systems, particularly in organometallic complexes or other bonding interactions. This index was calculated using the independent Gradient model (IGM). This approach focuses more on the electron density and its gradient, allowing for a more refined analysis of weak and strong interactions within the molecule. In adsorption investigation, it was stated that higher is IBSI value, strongest is the bond between metal and ligand.^39^

## 3. Results and discussion

### 3.1. Characterisation of the *Cystoseira sedoide*

**Fig. 1:**
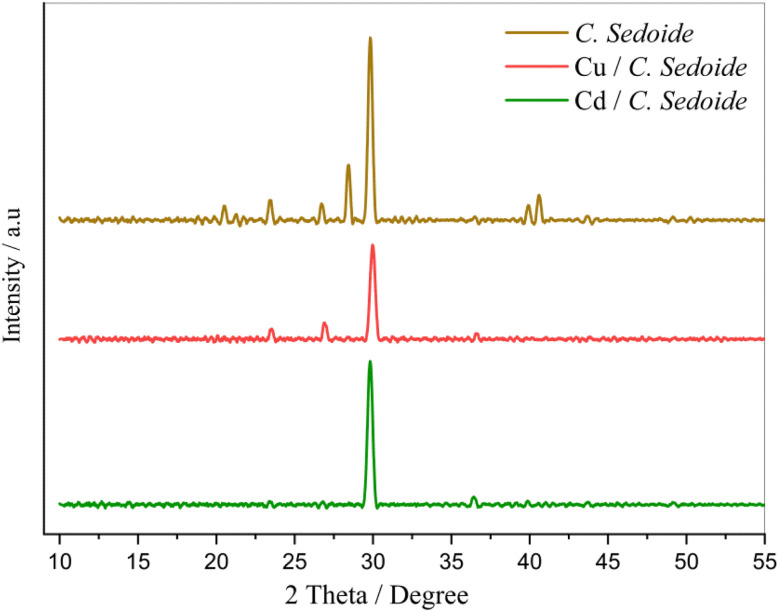
XRD patterns of *C. sedoide* powder before (brown curve) and after Cd^2+^ (green curve) and Cu^2+^ (red curve) biosorption.

Fig. 1 illustrates the XRD characterization spectra of *Cystoseira sedoide* powder before and after being mixed with cadmium and copper solutions. All XRD patterns exhibited a sharp peak at approximately 30° but a noticeable difference is revealed through the three patterns depicting a change in the alga morphology. A slight shift in the peaks was observed for *C. sedoide* powder mixed with copper solution, accompanied by peaks disappearance at 21°, 28°, 41°, and 42°. This can be attributed to the Cu ions biosorption process which might be ascribed to the pores surface coverage.^40,41^ However, the peaks observed at 21°, 23°, 26°, 28°, 41°, and 42° in the XRD pattern of the *Cystoseira sedoide* disappeared upon exposure to the cadmium solution, suggesting a significant interaction between the alga and the studied heavy metal ions. This difference in the biosorption capacity of *C. sedoide* towards Cd^2+^ and Cu^2+^ ions could be primarily attributed to the difference in metals affinity to the studied alga.^42^ However, it can be noted a decrease in the peak intensity at 28° for patterns of *C. sedoide* after the biosorption process of both Cd^2+^ and Cu^2+^, as well. The intensity of each peak reflects the number of diffracting planes contributing to the reflection of X-rays. It appears that the metal ions have obscured the crystalline plane. It is noticeable that the decrease in intensity differs upon the targeted heavy metal. The red line patterns related to *C. sedoide* after adsorption of copper ions presents the less intense peak revealing a more masking action of copper in comparison to cadmium ions.

**Fig. 2:**
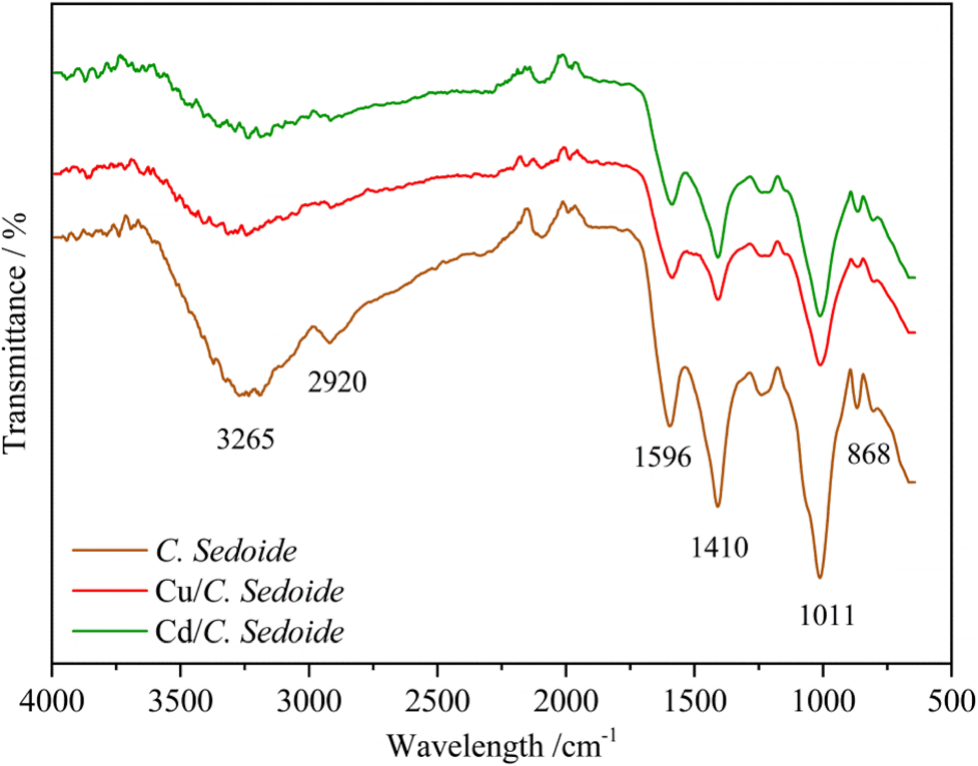
FTIR spectra of *C. sedoide* powder before (brown curve) and after Cd^2+^ (green curve) and Cu^2+^ (red curve) biosorption.

*Cystoseira sedoide* alga FTIR spectra before and after copper and cadmium biosorption are shown in Fig. 2. It can be observed, at first, that the three spectra have many similarities and many peaks occur in the same wavenumber region.

The functional groups of *C. sedoide* alga are in agreement with alginates spectra previously reported,^43^ given that they are predominantly present in brown alga. The main peaks can be listed as: a broad peak at 3265 cm^−1^ supported the presence of OH stretching groups related to the hydroxyl or carboxyl groups of the polysaccharide.^44,45^ The small peak at 2920 cm^−1^ was attributed to the symmetric CH stretching vibrations (*ν*CH, alkyl). The observed bands at 1596 cm^−1^ and at 1410 cm^−1^ attributed to asymmetric and symmetric stretching vibrations of the COO– groups, respectively, and are specific to the ionic binding.^46^ The shoulder at 1089 cm^−1^ relating to the C–C and C–O stretching can be also attributed to the presence of cross-linking.^47^ While the most intense peak at 1011 cm^−1^ refers to the C–O bonds of saccharide structure as well as the presence of guluronic acids^48^ and to the stretching vibrations of the glycoside bridge (C–O–C).^49^ The band observed at 868 cm^−1^ is attributed to out-of-plane C–H bending vibrations in aromatic groups, such as those found in phenolic compounds. Alternatively, it may also be related to sugar derivatives or secondary metabolites within the algal biomass, possibly linked to aromatic or modified carbohydrate structures.^50^

In the other hand, when heavy metals were biosorbed on *C. sedoide* alga, some changes in the respective spectra were observed. For instance, the peaks at 3265 cm^−1^, 1596 cm^−1^, 1410 cm^−1^, and 1011 cm^−1^ were remarkably lowered and sometimes slightly displaced; Table S1.† As a first approach, it can be suggested that the presence of heavy metals typically alters the O–H stretching vibration due to their interaction with the hydroxyl groups, leading to shifts, broadening, or intensity changes in the 3265 cm^−1^ peak. Cd^2+^ and Cu^2+^ bind to the carboxylate groups (COO^−^) of the alginate, causing a shift in the COO^−^ stretching vibration reflecting increased rigidity or a change in the conformation of the alginate polymer chains.^51^ The frequencies of these bands are highly sensitive to the structure of the carboxylate group, the nature of the solvent, the nature of the ligand and the involved metal ion.^52^ The bands related to carboxylate groups can be used as useful bands to follow the changes in the structure of different algae.^53^

### 3.2. Batch adsorption experiments

Different parameters were studied in order to achieve the optimal adsorption conditions. Experiments were also carried out onto the industrial activated carbon for an adequate comparison.

#### 3.2.1. Effect of adsorbent dose

**Fig. 3:**
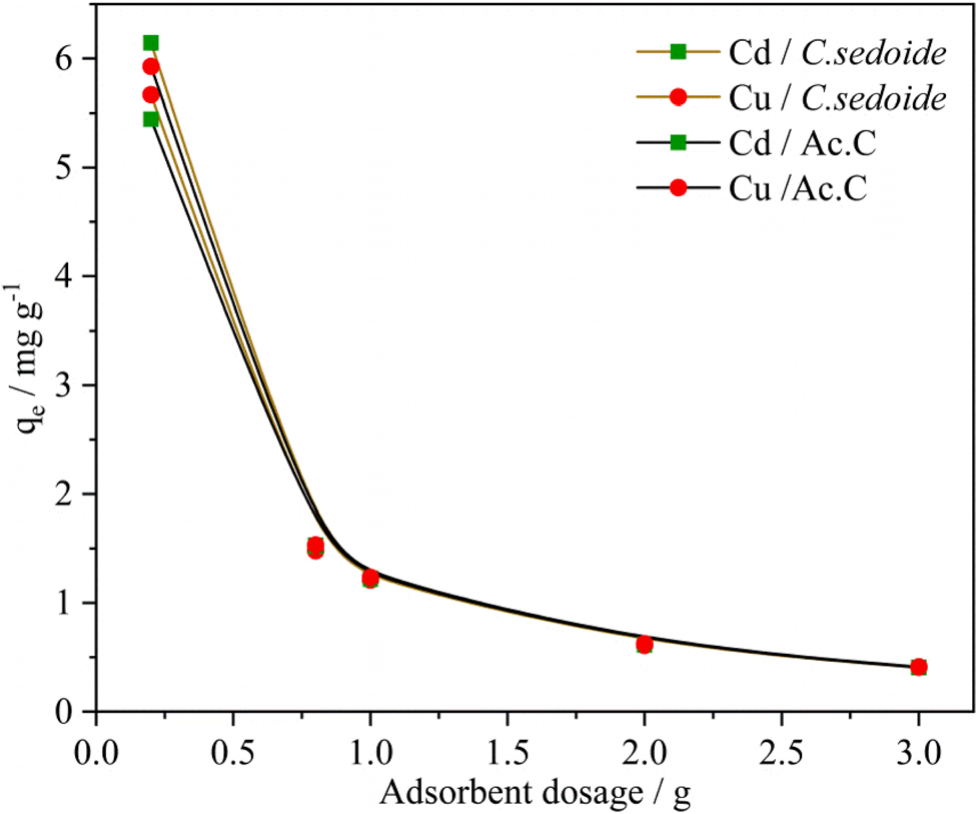
Effect of *C. sedoide* alga and Ac.C doses on the adsorption capacity of Cd^2+^ and Cu^2+^ ions.

The effect of alga dose on the copper and cadmium biosorption on the range [0.2 g; 3 g] was investigated by mixing the *C. sedoide* powder (0.5 g) with 25 mL of either Cd^2+^ or Cu^2+^ (50 mg L^−1^) solution. The contact time was set to 180 min under stirring at 300 rpm at room temperature. The same conditions were performed using Ac.C. As shown in Fig. 3 and S5,† a dose-removal relationship is evident for both Cd^2+^ and Cu^2+^. The results indicate that increasing the adsorbent dose enhances the removal rate but reduces the adsorption capacity. The removal rate reaches a maximum of 98.28% and 97.85% for Cd^2+^ and Cu^2+^, respectively, on *C. sedoide*. However, the adsorption capacity decreases significantly, dropping from 6 mg g^−1^ to 0.4 mg g^−1^ for all the adsorbents. This could be ascribed to the extent of available active sites for metal ions inherent to the surface functional groups. However, it is worth noting that the biosorptive removal of Cd^2+^ on alga follows a *quasi*-steady curve whatever was the biosorbent dose and varies slightly from 98.29% to 96.14% on the chosen dose interval range (Fig. S5†). This is probably due to the formation of agglomerates as alginates tend to form a gel in contact with water.^54^ On the other hand, cadmium and copper removal rate curves shape on Ac.C were different from those related to the *Cystoseira sedoide* and are more in agreement with other reported papers.^55^ In fact, *R*% usually increases with adsorbent dose till attaining a specific adsorbent dosage value then it remains almost constant.^56^ Despite the fact that the removal rate of copper ions on *C. sedoide* alga was above 90%, it seems that this latter behave differently with the both heavy metal ions and offers a better efficiency in the cadmium removal. This is an excellent feature since cadmium is recognized by far as more toxic than copper.

#### 3.2.2. Effect of pH

For the evaluation of the solution pH effect on the biosorptive removal of cadmium and copper ions, 0.5 g of the adsorbents were mixed with 25 mL of Cd^2+^or Cu^2+^ solution (50 mg L^−1^) where the solution pH was beforehand varied from 2.20 to 8.18 using HCl (1 M) or NaOH (1 M). The solution stirring was maintained up to 180 min with a stirring speed of 300 rpm.

**Fig. 4:**
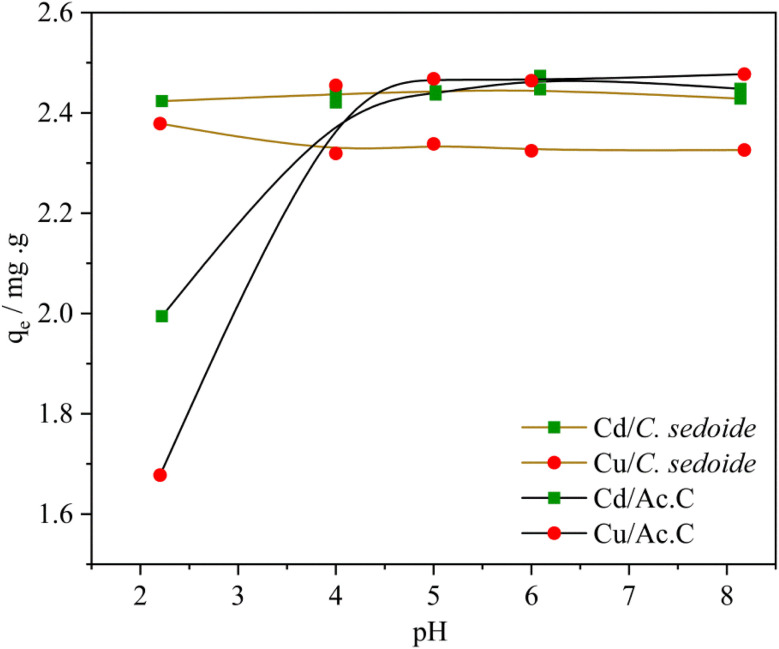
Effect of solution pH on the adsorption capacity of Cd^2+^ and Cu^2+^ ions by *C. sedoide* and Ac.C.

The obtained results showed that while copper and cadmium removal onto the activated carbon is dependent on the solution pH, the solution pH value has barely no effect on the removal, Fig. 4, and adsorption capacity, Fig. S5,† of the studied toxic metals by *Cystoseira sedoide*. In fact, the value of the adsorption capacity is kept constant around 2.4 mg g^−1^. The removal rate was almost equal to 97% and 93% for Cd^2+^ and Cu^2+^, respectively, on the alga. But, the heavy metal ions removal rate on the industrial activated carbon was low at acid pH and increased slowly to achieve a steady state above pH = 5 and beyond at alkali pH solutions.^55^

The attained steady state could be explained by the transformation of metal ions into their respective hydroxides at higher pH values as shown by *E*-pH diagrams for copper and cadmium.^57^

#### 3.2.3. Effect of initial Cd^2+^ and Cu^2+^ concentration

**Fig. 5:**
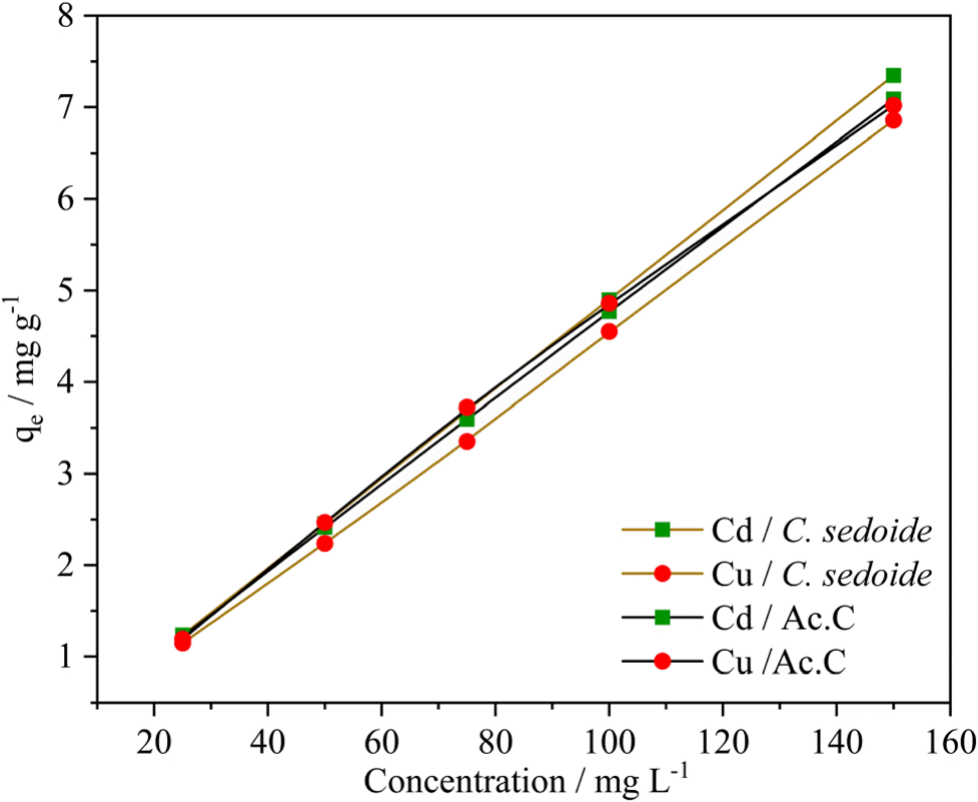
Effect of Cd^2+^ and Cu^2+^ ions concentration on their adsorption capacity by *Cystoseira sedoide* and Ac.C.

Knowing that the metal ions concentration is a major parameter which usually affects heavy metals elimination, this study was carried out in the aim to determine to what extent it impact the adsorption process. Metal ions concentration was varied from 25 to 150 mg L^−1^ while the other parameters were maintained constant (stirring speed: 300 rpm, adsorbent dose (alga/Ac.C): 0.5 g, contact time: 180 min, and solution volume: 25 mL). The adsorption curves are gathered in the subsequent Fig. 5.

An increase in the adsorption capacity was observed upon the metal ions concentration increase, Fig. 5. A maximum *q*_e_ of 7.35 and 6.85 mg g^−1^ was achieved at 150 mg L^−1^ for Cd^2+^and Cu^2+^, respectively. Furthermore, it appears from Fig. S5† that Cd^2+^ and Cu^2+^ removal rates show an almost steady state as function of their concentration with a mean value at 97% for cadmium ions and 85% for copper ions. The alginate responsible for the heavy metal biosorption encompasses six hydroxyl groups in addition to the carboxyl group which are all prone to form bonds with heavy metals. The plenty of functional groups is apparently responsible for, not only, the excellent adsorptive removal of *Cystoseira sedoide* for Cd^2+^ and Cu^2+^ ions, but also the insensitivity of this alga to the increase of ions concentration. This means that *Cystoseira sedoide* alga has an unexpected ability to eliminate the heavy metal ions at a point source pollution (which is characterised in general by high levels of pollution), as well as in the case of a chronic pollution. However, copper and cadmium removal curves followed a different shape on the activated carbon. Indeed, heavy metals removal rate was high at lower values of the initial concentrations and exhibited lesser values of the removal rate as the initial concentration of copper, as well as that of cadmium grew. This feature is most often encountered during the adsorption phenomena.^35,55^ As explained by many authors, this is due to an adequate proportion of the available active sites to the heavy metal ions at lesser values of the initial concentrations. Nevertheless, this favourable ratio is more and more affected by the rise in ions concentration. This was imputed to the significant number of Cu^2+^ and Cd^2+^ ions that might lead to a screening phenomena of the negative charge and a saturation of the active sites.^58^

#### 3.2.4. Adsorption equilibrium studies

The most employed adsorption isotherms, namely; Langmuir, Freundlich, and Temkin were applied in this study to provide the maximum adsorption capacities and to give a correct description of the phenomena.

The non-linearized Langmuir equation is as follows:^35,59^9
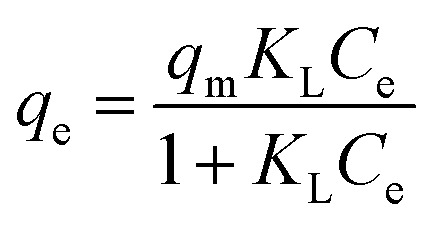
where; *q*_m_ is the maximum adsorption capacity (mg g^−1^) and *K*_L_ is the affinity constant (L mg^−1^).

From Langmuir isotherm, a dimensionless parameter; *R*_L_, can be extracted to qualify the favourability of the studied heavy metal ions adsorption onto the adsorbent. *R*_L_ is as follows:^60^10
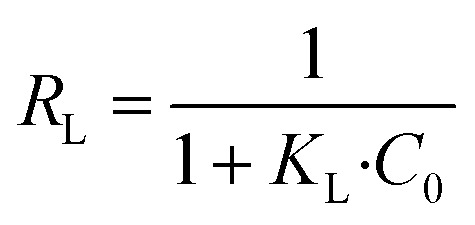


The non-linearized Freundlich and Temkin equations are as follows:^11,59^11
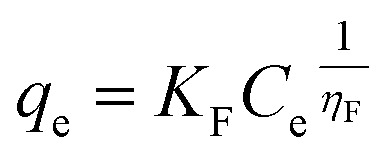
12
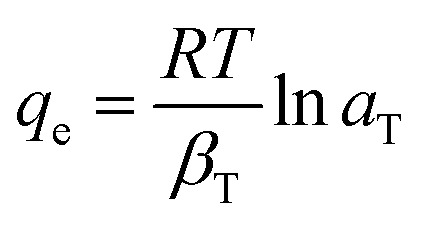
where; *K*_F_(mg g^−1^)(L mg^−1^) and 1/*η*_F_ are Freundlich constants which reflect the intensity and the adsorption capacity, respectively, *β*_T_ is the Temkin constant related to the adsorption heat (J mol^−1^), and *a*_T_ is the Temkin isotherm constant (L g^−1^).

**Fig. 6:**
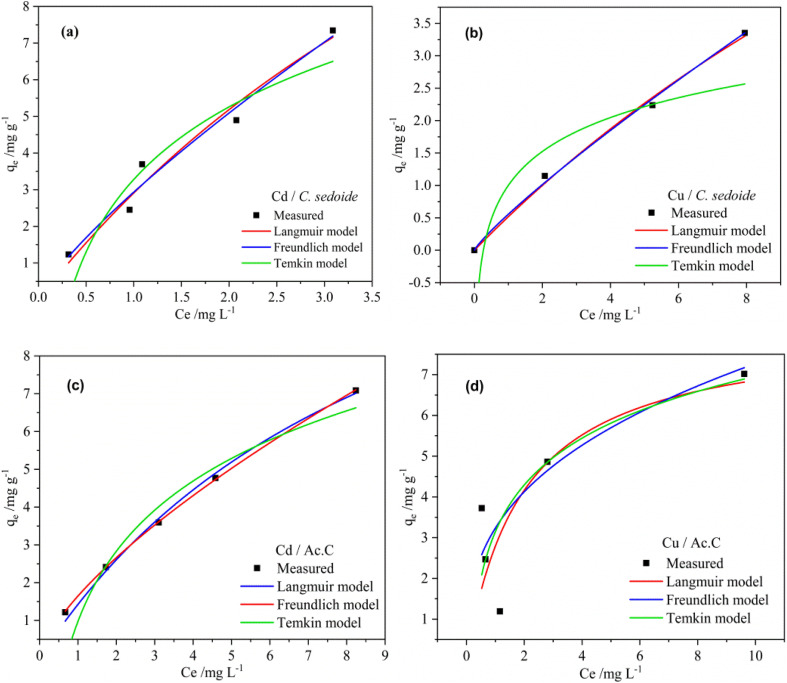
Fitting of the adsorption isotherms for Cd^2+^ and Cu^2+^ ions using *Cystoseira sedoide* (a and b) and the industrial activated carbon (c and d).

As depicted by the fitting of the adsorption isotherms in Fig. 6, and the corresponding calculated parameters appearing in Table 1, it seems that Freundlich isotherm model provides the best fit for the studied metal ions on *C. sedoide* and Ac.C.

**Table 1:** Langmuir, Freundlich, and Temkin parameters for Cd^2+^and Cu^2+^ adsorption onto *C. sedoide* and the commercial activated carbon

Isotherm	Parameters	Cd^2+^	Cu^2+^	Cd^2+^	Cu^2+^
		*Cystoseira sedoide*		Activated carbon	
Langmuir	*R* ^2^	0.967	0.995	0.9951	0.622
	*K*_L_ (L mg^−1^) *q*_m_(mg g^−1^)	0.1394	0.0366	0.1025	0.5170
	*R*_L_	23, 78	14.66	15.31	8.19
		0.125	0.353	0.163	0.037
Freundlich	*R* ^2^	0.9727	0.9993	0.998	0.704
	*K*_F_ (mg g^−1^)(L mg^−1^)^*η*_F_^	2.9345	0.5555	1.6466	3.2335
		1.2573	1.1530	1.4424	2.8416
Temkin	*R* ^2^ *a*_T_ (L g^−1^)	0.888	0.7973	0.9743	0.644
	*β*_T_ (J mol^−1^)	4.026	3.7674	1.4379	6.6774
		2.8573	0.7545	2.6811	1.6563

The calculated maximum adsorption capacities were, respectively, 23.78 mg g^−1^ and 14.66 mg g^−1^ for Cd^2+^ and Cu^2+^ ions on *Cystoseira sedoide* and 15.31 mg g^−1^ and 8.19 mg g^−1^ for cadmium and copper ions on the commercial activated carbon. Moreover, the calculated dimensionless parameter *R*_L_, which was less than unity in all cases, indicated that the adsorption of Cd^2+^ and Cu^2+^ is favorable either on *C. sedoide* alga or onto Ac.C.

From the results, it is obvious that Cd^2+^ and Cu^2+^ behave differently depending on the adsorbent. The ions were biosorbed according to a multilayer configuration onto alga, due to a non-uniform levels of energy through the active sites.^61^ This is a common and an expected result for heavy metals on activated carbons.^35^ On the other hand, the *η*_F_ factor translates a feasible adsorption process.

A close comparison of Cd^2+^ and Cu^2+^ removal by different algal biosorbents, Table 2, showed that the maximum adsorption capacity of *C. sedoide* was often greater than those reported by the literature.

**Table 2:** Adsorbents capacities of various algal biosorbents for cadmium and copper biosorption from aqueous solutions

Metal	Biosorbent	*C*_0_ (mg L^−1^)	*q*_m_ (mg g^−1^)	Ref.
Cd^2+^	*Nizamuddinia zanardin*	118.0	4.04	62
	*Padina australis*	118.0	3.49	62
	*Sargassum gluacescen*	118.0	4.23	62
	*Cystoseira indica*	118.0	8.35	62
	*Neochloris oleoabundans*	50.0	73.06	63
	*Mastocarpus stellatus*	34.0	7	64
	*Cystoseira sedoide*	50.0	23.78	Present study
Cu^2+^	*Neochloris oleoabundans*	50.0	78.16	52
	*Fucus vesiculosus*	20	11.1	65
	*Chaetomorpha antennina*	20	25.78	66
	*Ulva lactuca*	60	62.5	67
	*Vaucheria*	250	46.29	68
	*Codium vermilara*	10–150	16.9	69
	*Spirogyra insignis*	10–150	19.3	69
	*Cystoseira sedoide*	50.0	14.66	Present study

#### 3.2.5. Effect of the contact time

The contact time effect on the studied heavy metals elimination was conducted by mixing 50 mL of the metal solution (50 mg L^−1^) with 1.0 g of *C. sedoide* or Ac.C at room temperature with a stirring speed of 300 rpm. 0.5 mL was taken from the mixture at different contact times and the residual heavy metal ions concentration was measured using AAS.

**Fig. 7:**
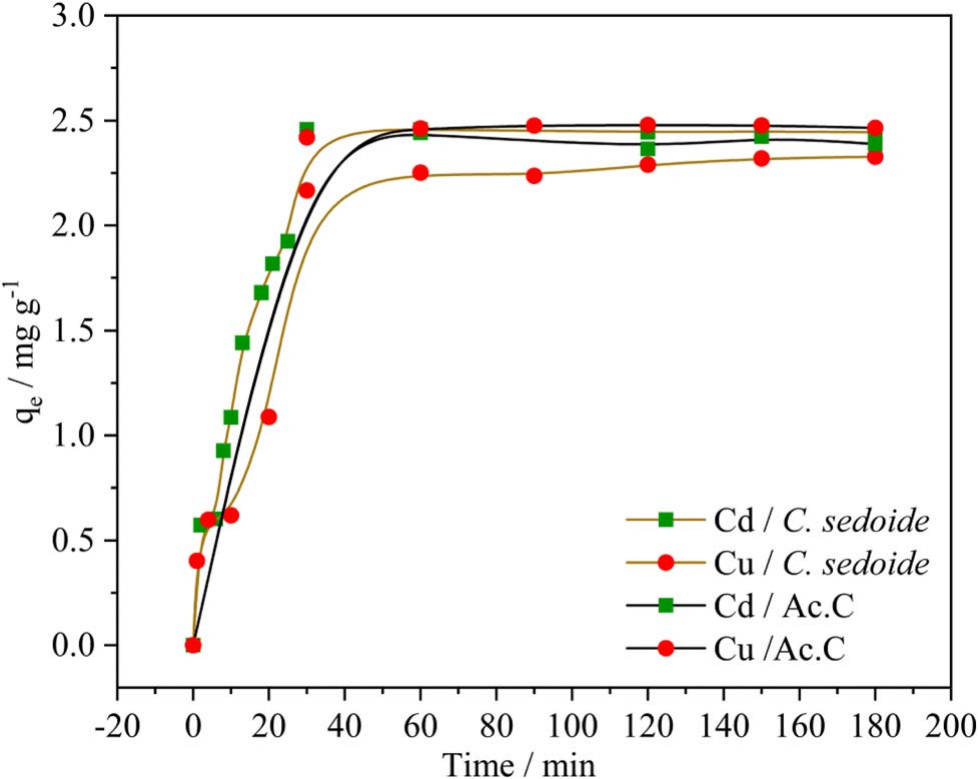
Effect of contact time on adsorption capacity of Cd^2+^ and Cu^2+^ on *Cystoseira sedoide* and on activated carbon.

Fig. 7 portrays heavy metal ions biosorption capacity variation upon time. This latter increases rapidly at the first 40 minutes where a maximum biosorption capacity was observed at around 2.4 mg g^−1^ for both adsorbents, corresponding to the following removal rates Fig. S5:† 98% and 83% for Cd^2+^ and Cu^2+^ onto *C. sedoide*, respectively, and 98% and 99% for Cd^2+^ and Cu^2+^ onto the commercial activated carbon, respectively. A very slow biosorption kinetics followed the first step and a steady state is then observed where the removal rate didn't change that much. As stated by other authors, the rapid biosorption step is commonly related to the available active sites on the *C. sedoide* alga surface.^69^ After a while, the biosorption sites become less available because of their occupancy, and by the way, the removal rate diminishes and achieves a steady state. It is worth noting that the maximum removal rate for Cd^2+^ ions onto *C. sedoide* and Ac.C after 40 min is around 99%, same for copper onto Ac.C. However, Cu^2+^ biosorption by *Cystoseira sedoide* is about 8% lesser than that on Ac.C. From the above results, it appears that *C. sedoide* has a better affinity for Cd^2+^ retention than that for Cu^2+^ ions. This phenomenon was reported earlier.^70^

#### 3.2.6. Adsorption kinetics studies

Two kinetics models, namely the pseudo-first order and the pseudo-second order were used to fit the experimental data. The non-linearized equations of the pseudo-first order and the pseudo-second order are given by the following expressions:^71^

13 *q*_t_ = *q*_e_(1 − e^*K*_i_*t*^)

14
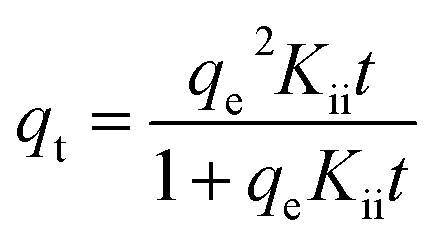
where; *K*_i_ (min^−1^) and *K*_ii_ (g mg^−1^ min^−1^) are, respectively, the kinetics constants of the pseudo-first order and the pseudo-second order.

Table 3 and Fig. S6† suggest that the pseudo-second order appears as more appropriate for the fitting of the experimental findings (*R*^2^ > 0.92). The very good agreement between the experimental equilibrium adsorption capacities (*q*_e.exp_) and the calculated ones (*q*_e.cal_) supports once again the pseudo-second order kinetics model. However, it can be noted that the kinetics constants *K*_ii_ for the elimination of Cd^2+^ and Cu^2+^ ions by *C. sedoide* alga are very close which means that notwithstanding their different physicochemical properties such as size and weight, these ions have similar velocities indicating that the slight discrepancies between the adsorption processes for the both metal ions are more likely related to a higher affinity of the *Cystoseira sedoide* to Cd^2+^. In the other side, the calculated kinetics constants for the adsorption of Cd^2+^ and Cu^2+^ on the activated carbon are greater than that on the alga.

**Table 3:** Kinetics parameters for the removal of Cd^2+^ and Cu^2+^ using *Cystoseira sedoide*

Kinetics model	Parameters	Cd^2+^	Cu^2+^
Pseudo-first order	*R* ^2^	0.958	0.906
	*K*_i_ *q*_e.cal_	0.065	0.045
		2.349	2.30
Pseudo-second order	*R* ^2^	0.969	0.920
	*K*_ii_ *q*_e.cal_	0.030	0.023
	*Θ*	2.650	2.307
		0.210	0.122
Experimental result	*q*_e.exp_	2.450	2.240

The initial adsorption rates; *Θ* (mg g^−1^ min^−1^), were calculated using the following expression;^72^

15 *Θ* = *K*_ii_*q*_e_^2^

*Θ* Values indicated that the studied heavy metal ions biosorption on *C. sedoide* alga is broadly slower than that on the activated carbon and on other adsorbents reported in the literature;^61^ thus indicating a typically slow biosorption kinetics. However, it appears that it doesn't affect the overall biosorption process.

According to the postulated definition of the pseudo-second order, the limiting step is the chemisorption which highlights the importance of interactions between biosorbent and metal ions in the solution.^73^

Dubinin–Radushkevich model was also applied for the fitting of the kinetics data (Table S2†). The determination coefficient for this model was high but not enough to consider it as the more relevant. However, the model allowed the calculation of the mean free energy of biosorption (*E*).^59,74^ All mean free energies of biosorption were ranged between 8.45 kJ mol^−1^ and 15.58 kJ mol^−1^ denoting a chemical biosorption. This is in agreement with the above results.

#### 3.2.7. Temperature effect and thermodynamic study

This investigation was performed by varying the temperature from 20 to 60 °C. A constant adsorbent mass; 0.5 g of *Cystoseira sedoide* or of activated carbon, was mixed with 25 mL of the heavy metal solution (50 mg L^−1^). The contact time was 180 minutes under solution stirring at 300 rpm.

**Fig. 8:**
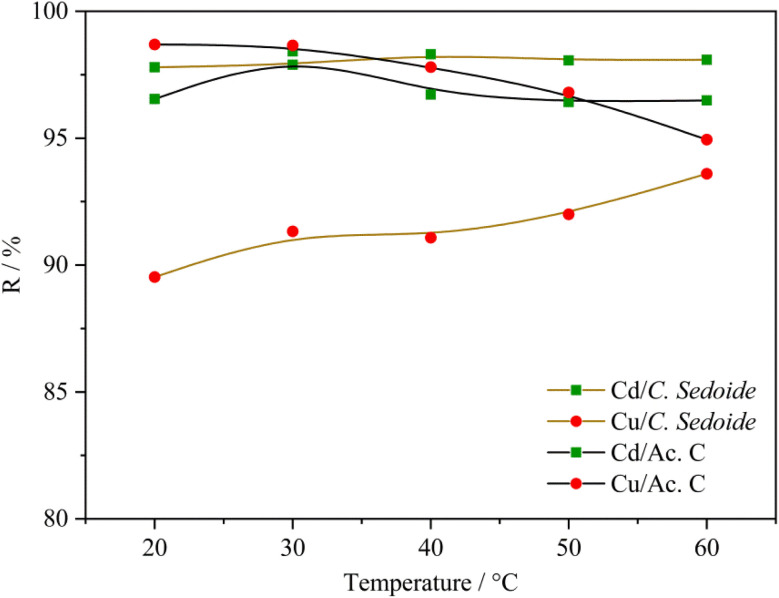
Effect of the temperature on the removal rate of cadmium and copper ions by *Cystoseira sedoide* and activated carbon.

Fig. 8 portrays the change in the removal rate according to the temperature. It can be stated that the studied heavy metals elimination on *C. sedoide* is not temperature-dependant. Indeed, the removal rate increases by 4% and 0.3% for Cu^2+^ and Cd^2+^, respectively, when temperature was raised from 20 °C to 60 °C. A slight difference of the temperature effect can be observed dependent on the implied heavy metal ion and the chosen adsorbent.

As reported by many papers, the adsorption phenomenon is always accompanied by a thermal process. While the exothermic adsorption process indicates that the interactions between the adsorbed molecules and the surface are weaker than the interactions in the original phase or that significant rearrangements must occur for adsorption to take place (physical adsorption), the endothermic process is revealing that the interactions between the adsorbed molecules and the surface are strong enough to release heat. This suggests that the process is thermodynamically favored and that the molecules are stabilized on the surface (chemical adsorption). This is why the determination of the thermodynamic parameters is crucial.^75^

The standard free Gibbs energy Δ*G*^0^, the standard enthalpy Δ*H*^0^, and the standard entropy Δ*S*^0^ can be expressed using the equations below:

16 Δ*G*^0^ = −*RT* ln *K*_d_

17
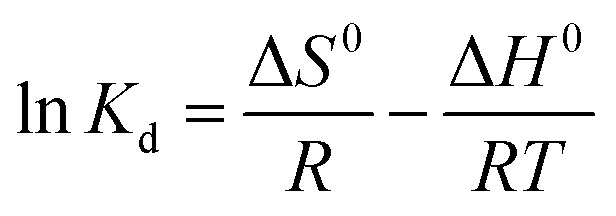
18
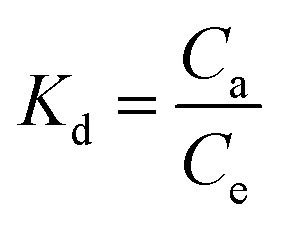


**Fig. 9:**
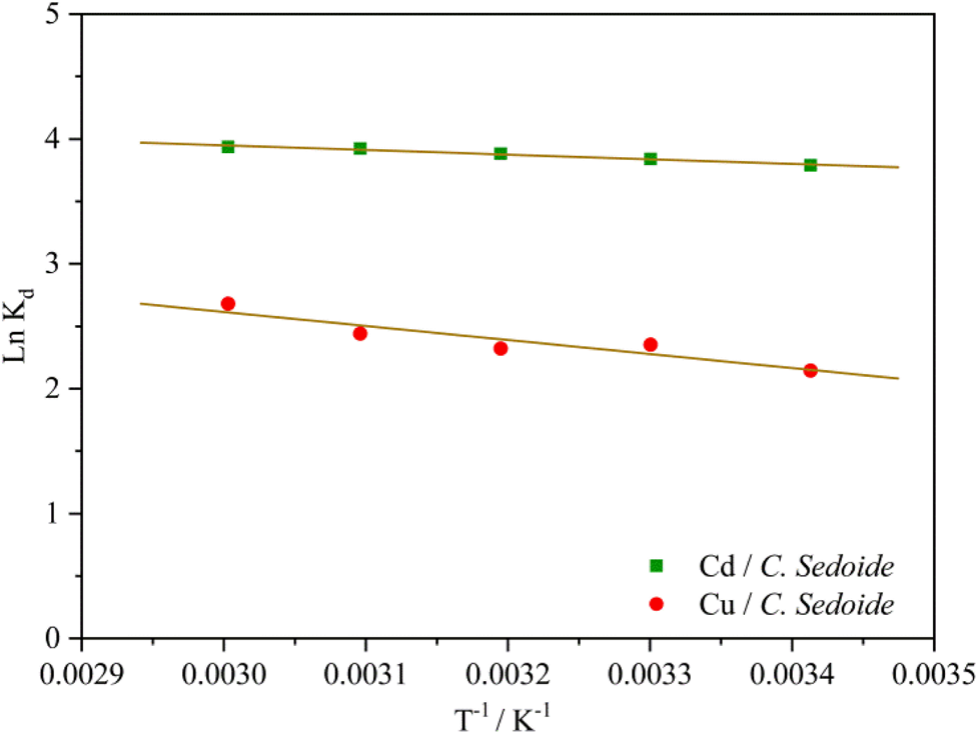
Thermodynamic curves of Cd^2+^ and Cu^2+^ biosorption on *Cystoseira sedoide* alga.

The plot of the ln *K*_d_ as a function of the reverse of temperature gave the thermodynamic parameters by extracting the plot slop and intercept; Fig. 9.

Fig. 9 and Table S3† showed that the standard Gibbs free energy change Δ*G*^0^ was negative whatever was the operating temperature. Whereas, the sign of the change in the standard enthalpy reflected once again an endothermic process which means that the raise in temperature favored the heavy metal ions biosorption.

#### 3.2.8. Cadmium biosorption in the presence of copper

Results related to cadmium biosorption by *Cystoseira sedoide* alga in the presence of copper were conducted by measuring the biosorption capacity through 120 min and employing the previously used isotherms to model the biosorption equilibrium. Similarly to single ion biosorption experiments, it was found that Langmuir isotherm fits well the co-biosorption data, Fig. S7 and Table S4.† The calculated competitive biosorption parameters, in this case; the biosorption capacity ratio (*q*_ratio_) and biosorption reduction rate (Δ*Y*) revealed that Cd^2+^ ions are relatively affected by the co-occurrence of copper ions in the same solution. This may be explained by strong interactions between Cu^2+^ and alga, implying, apparently, that they have bonded differently than Cd^2+^.

## 4. Desorption assays

The desorption study was conducted onto the *Cystoseira sedoide* alga used after the single biosorption of cadmium and after the competitive biosorption of Cd^2+^ in the presence of Cu^2+^. Results showed that Cd^2+^ recovery in nitric acid solution (1%) was about 40% in single metal configuration and 8.5% in the presence of Cu^2+^. It was reported earlier that metal ion desorption is less efficient in multi-metal systems.^59,76^ Furthermore, the chemisorption of cadmium ions onto *C. Sedoide* implied strong interactions that cannot be broken with a such low concentration acid. These findings are encouraging since it is preferable to obtain cadmium ions in a solid state rather than in an acid aqueous solution. Moreover and regarding the results, it seems that cadmium ions should not be leached by acid rain if improperly stored. However, in the aim of a cadmium ions recovery, it's quite easy to retrieve the metal as an added value to this elimination by the use of a more acidic solution.^61^

## 5. DFT calculations

### 5.1. Molecular electrostatic potential (MEP)

Electrostatic potential maps visualize the distribution of electron density across a molecule. They show where a molecule is likely to donate or accept electrons based on the electron density.^77^

**Fig. 10:**
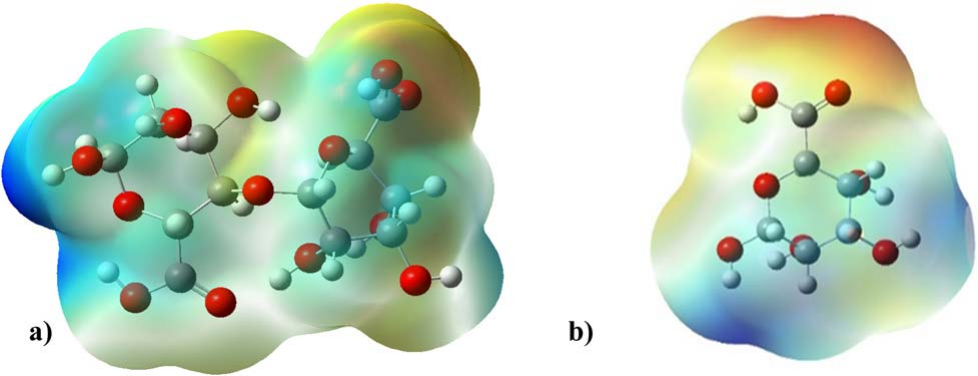
MEP on the isodensity surface of (a) ALG (monomer) and (b) ALG (dimer).

The molecular electrostatic potential of both ALG (monomer) and ALG (dimer) was employed to elucidate the local reactivity of the molecules. Areas marked in intense orange or red hues denote regions with the highest negative charge density, suggesting these sites are most susceptible to electrophilic attack. Conversely, regions shown in deepest blue represent the highest positive charge density, indicating favorable sites for nucleophilic attack. The optimized structure and MEP of the proposed structures of alginate were presented in Fig. 10. The figure reveals that the most significant positive charge density is concentrated on the hydrogen of the hydroxyl group, identifying these as prime sites for nucleophilic interactions. The most negative region, indicated in red, is situated on the oxygen atom of the carbonyl group, marking the electrophilic sites.

**Fig. 11:**
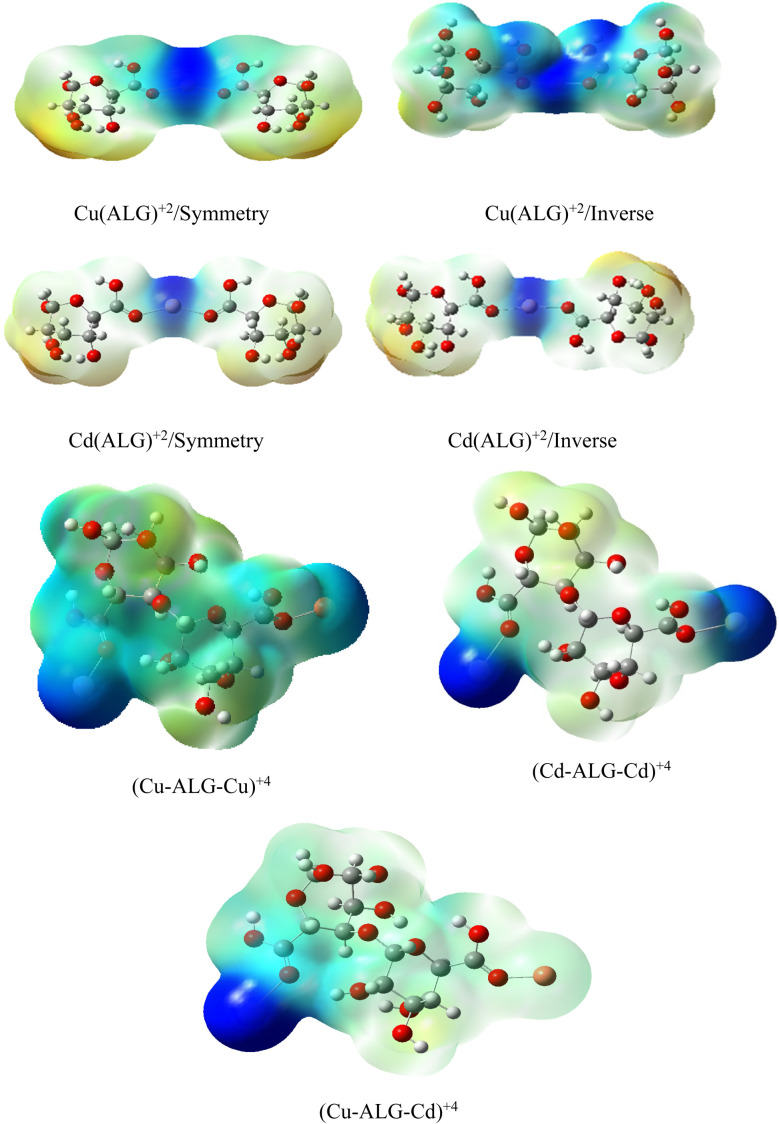
Molecular electrostatic potential of complexes at B3LYP-D3/LanL2DZ level. The surfaces were plotted by the 0.0004 electrons/b^3^ contour of the electronic density.

After interaction, the MEP maps of Cu(ALG)^2+^/symmetry, Cu(ALG)^2+^/inverse, Cd(ALG)^2+^/symmetry, Cd(ALG)^2+^/inverse, (Cu-ALG-Cu)^4+^, (Cd-ALG-Cd)^4+^, (Cu-ALG-Cd)^4+^ complexes (Fig. 11) show a concentration of dark blue color on the metal atoms which suggests that they are positively charged or are electron-deficient. They also show that the interaction regions are located on the blue regions where the metal ion is interacting with the electron-rich oxygen atom of carbonyl group of the alginate, facilitating the formation of stable complexes. This is consistent with the fact that metals (especially divalent ones like Cd^2+^ or Cu^2+^) tend to form bonds with negatively charged oxygen atoms in ligands.^39^ The oxygen atoms (with charges nearly from (−0.30 to −0.49)) are expected to have more negative electrostatic potential than other atoms in the structure. Indeed, the areas around the oxygen atoms show slightly negative regions, appearing as green and/or yellow. Light blue color on the copper ion on the MEP map of (Cu-ALG-Cd)^4+^ complex means that copper is receiving more electron density from the ligand, making it less electropositive compared to cadmium. A higher charge transfer would improve copper's biosorption efficiency since it can more readily interact with the ligand's electron-donating sites, such as oxygen atoms in the carbonyl groups. The dark blue color shows that cadmium remains more electropositive and less capable of charge transfer. This implies that cadmium is less involved in charge-sharing interactions with the ligand, resulting in weaker biosorption efficiency. The high positive charge on cadmium may limit its interaction with the carbonyl oxygen, leading to a more electropositive and less reactive environment compared to copper.

### 5.2. HOMO and LUMO energy analysis

The theory of molecular boundary orbitals postulates that the Highest Occupied Molecular Orbital and the Lowest Unoccupied Molecular Orbital are crucial determinants of a molecule's reactivity. Essentially, these orbitals enable the prediction of the most reactive sites within a system and facilitate the analysis and elucidation of various reaction mechanisms. This concept, known as frontier orbitals theory, is integral to Density Functional Theory. A higher energy level of the (HOMO) in a molecule correlates with an increased propensity for electron loss, thereby accelerating its electron-donating reactivity. Conversely, a lower energy level of the (LUMO) signifies an enhanced capacity for electron acceptance.

**Fig. 12:**
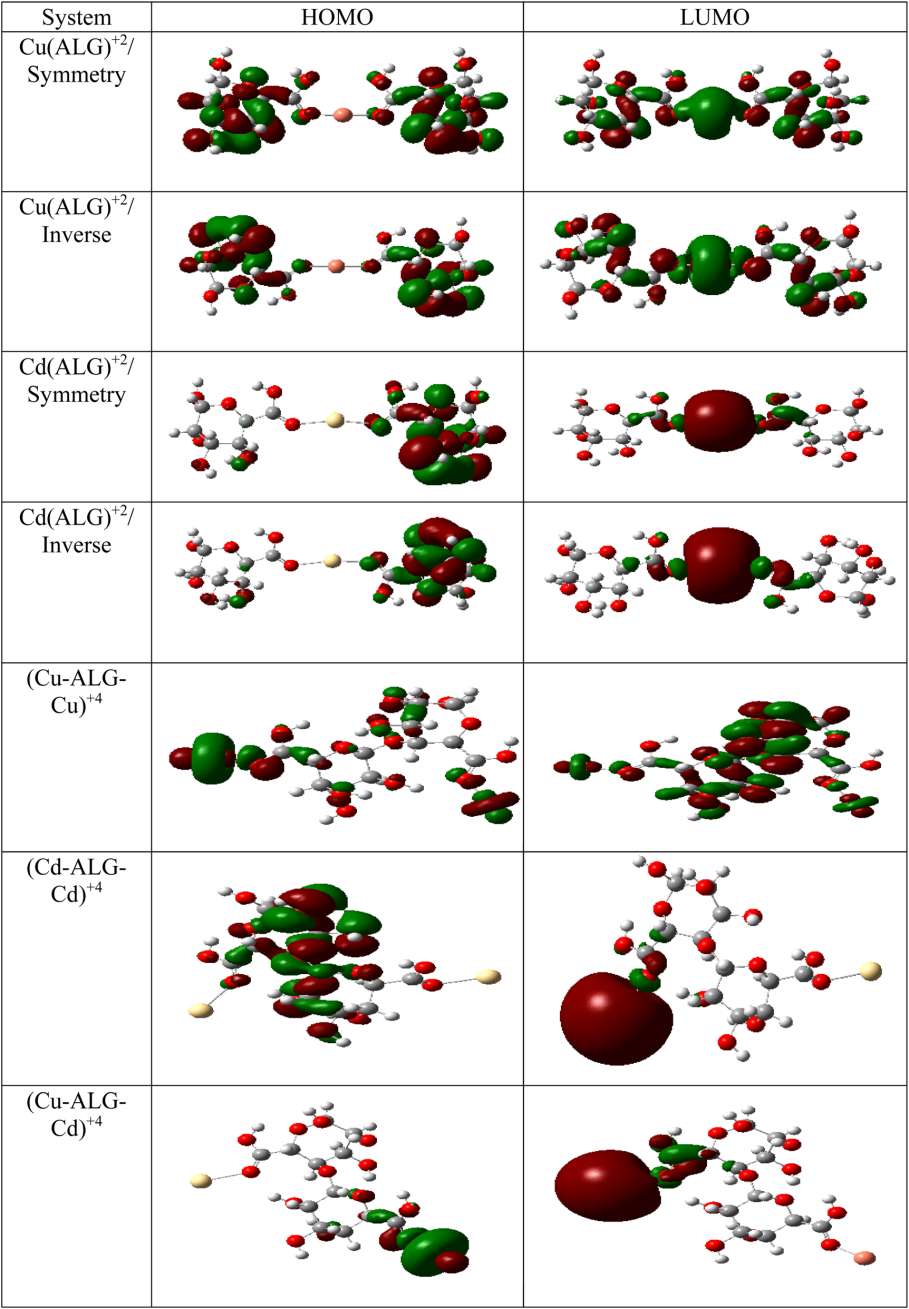
HOMO and LUMO energies of Cu(ALG)^2+^, Cd(ALG)^2+^, (Cu-ALG-Cu)^4+^, (Cu-ALG-Cd)^4+^ and (Cd-ALG-Cd)^4+^.

The energy difference between the HOMO and LUMO is commonly referred to as the HOMO–LUMO energy gap, denoted as Δ*E*. The energy gap, can be utilized to predict the relative strength and stability of chemical species. A reduced energy gap implies enhanced reactivity, which may translate to a greater capacity for metal biosorption. Such a complex possesses a higher availability of electrons, facilitating more effective interaction with metal ions and thereby strengthening adsorption. In adsorption research, complexes with narrower energy gaps typically exhibit increased electronic flexibility, which enhances their interaction with the adsorbate and results in more efficient adsorption. The HOMO and LUMO results of Cu(ALG)^2+^/symmetry, Cu(ALG)^2+^/inverse, Cd(ALG)^2+^/symmetry, Cd(ALG)^2+^/inverse, (Cu-ALG-Cu)^2+^, (Cd-ALG-Cd)^2+^, (Cu-ALG-Cd)^2+^ complexes are presented in Fig. 12 and their energies are summarized in Table 4. From Table 4, we find that for both copper and cadmium complexes, the energy gap is marginally reduced in the symmetric configuration relative to the inverse and dimer configuration. This observation implies that the symmetry configuration may exhibit slightly greater reactivity. A quite comparison of the two metals reveals that the energy gap (Δ*E*) was lower for Cu complexes than for Cd complexes, indicating that Cu complexes possess greater reactivity, and strong interaction compared to Cd complexes. This result was confirmed by the lower hardness value of copper complexes.

**Table 4:** HOMO and LUMO orbital energies, along with global reactivity metrics

Property	*E*_LUMO_ (eV)	*E*_HOMO_ (eV)	Δ*E* (eV)	Hardness, *η* (eV)	Potential, *μ* (eV)	Electrphilicity *ω* (eV)
Cu(ALG)^2+^/symmetry	−3.3116	−8.2423	4.9307	2.4653	5.7797	0.0571
Cu(ALG)^2+^/inverse	−2.7293	−8.1443	5.4123	2.7048	5.4368	0.0517
Cd(ALG)^2+^/symmetry	−1.9701	−7.2572	5.8286	2.9143	4.8844	0.0462
Cd(ALG)^2+^/inverse	−2.0326	−7.8042	5.7715	2.8871	4.9198	0.0462
(Cu-ALG-Cu)^4+^	−2.5986	−7.4722	4.8735	2.4354	5.0368	0.0408
(Cd-ALG-Cd)^4+^	−2.6259	−7.6518	5.0259	2.5116	5.1375	0.0435
(Cu-ALG-Cd)^4+^	−2.6341	−7.6001	4.9660	2.4816	5.1184	0.0435

The chemical potential is slightly lower for cadmium complexes if compared to copper complexes, suggesting that the formers are less prone to electron donation or acceptance compared to Cu complexes. Electrophilicity (*ω*), which gauges the tendency to accept electrons, is also lower in Cd complexes than in Cu complexes. Lower *ω* values denote a tendency towards nucleophilicity, whereas higher *ω* values indicate greater electrophilicity. Consequently, the reduced electrophilicity of Cd complexes signifies a lower tendency to accept electrons in comparison to copper complexes and this is may explain that cadmium predominantly adopts the +2 oxidation state in its complexes, characterized by the electron configuration 4d^10^. This results in a completely filled 4d subshell for Cd^2+^, leading to a stable electronic arrangement that exhibits minimal propensity for additional electron acceptance. While, copper can be found in several oxidation states, including +1 and +2. In the +2 oxidation state, copper is configured as 3d^9^, where the d-subshell remains incomplete and thus less stable. This incomplete d-orbital configuration in Cu^2+^ makes it more prone to accept extra electrons in order to attain a more stable electronic state. Furthermore, there is a slight decrease in electrophilicity in the inverse and dimer configurations for both metals, implying that these configurations may exhibit a reduced tendency for electrophilic behavior.

From the findings, it appears that the stability and interaction properties of the proposed complexes are modulated by the metal type and the specific configuration of the complexes. The results indicate that Cu-ALG complexes exhibit moderately greater reactivity, demonstrate lower stability, and possess a stronger affinity to alginate and greater tendency to donate electrons relative to Cd-ALG complexes. Furthermore, the dimer configuration generally reflects improved reactivity and diminished stability when compared to the symmetry and inverse configurations, which could signify an enhanced capacity for metal absorption. The (Cu-ALG-Cu)^4+^ possesses more accessible electrons for engaging with copper ions, thereby facilitating more robust adsorption.

### 5.3. Effect of coordination geometry

To elucidate the interaction strength between copper-alginate and cadmium-alginate in both symmetric and inverse configurations, various interaction conformations were analyzed *via* DFT calculations. The findings are detailed in Table 5. A smaller complex energy and shorter bond length correlates with a higher IBSI and a more intense interaction force. The DFT calculation results indicated that the complex energies were ranked as follows:

**Table 5:** Complex energies, bond length and IBSI values of Cu (ALG)^2+^, Cd (ALG)^2+^, (Cu-ALG-Cu)^4+^, (Cd-ALG-Cd)^4+^, and (Cu-ALG-Cd)^4+^

Complex	Optimized structure	*E*_complex_ (Ha)	Bond length (Å)	IBSI
Cu (ALG)^2+^/symmetry	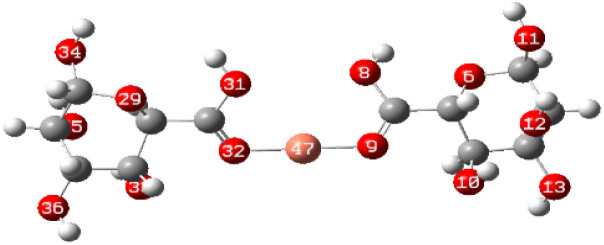	−1717.9626	Cu–O(9): 1.831	0.5471
			Cu–O(32): 1.829	0.5501
Cu (ALG)^2+^/inverse	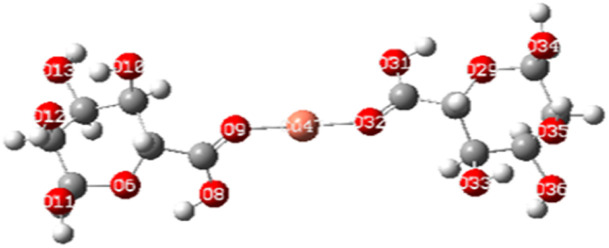	−1718.0716	Cu–O(9): 1.875	0.4850
			Cu–O(32):1.874	0.4851
Cd (ALG)^2+^/symmetry	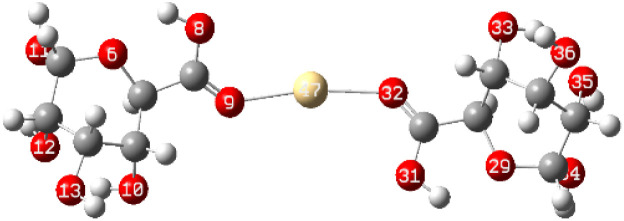	−1570.1307	Cd–O(9): 2.284	0.2424
			Cd–O(32):2.303	0.2320
Cd (ALG)^2+^/inverse	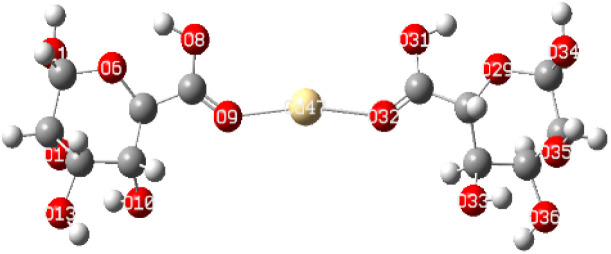	−1570.1335	Cu–O(9): 2.264	0.2542
			Cu–O(32): 2.278	0.2461
(Cu-ALG-Cu)^4+^	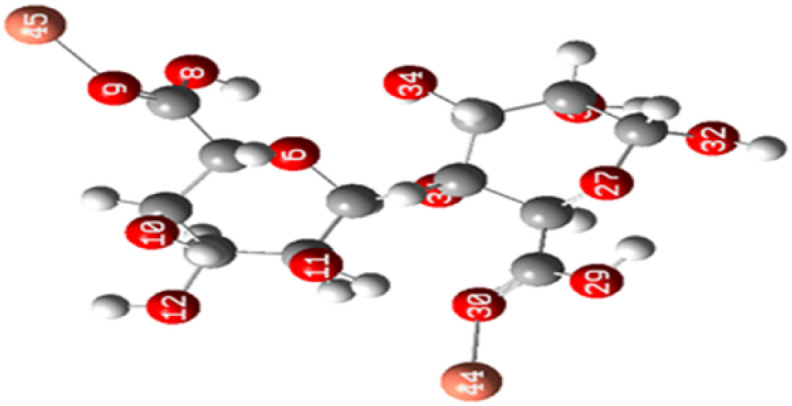	−1837.3837	Cu(45)–O(9): 1.9511	0.3958
			Cu(44)–O(30): 1.9557	0.3910
(Cd-ALG-Cd)^4+^	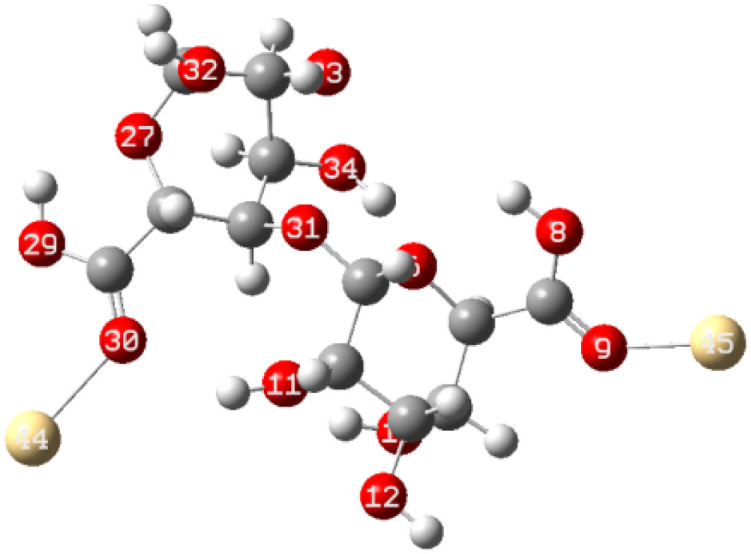	−1541.5219	Cd(45)–O(9): 2.3580	0.2040
			Cd(44)–O(30): 2.3014	0.2332
(Cu-ALG-Cd)^4+^	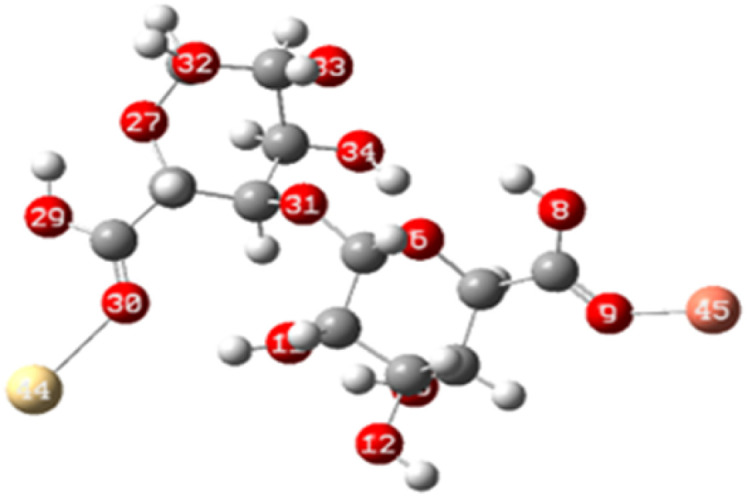	−1689.2020	Cd(44)–O(30): 2.3272	0.2194
			Cu(45)–O(9): 1.9487	0.3983

−1717.9626 Cu (ALG)^2+^ < 1570.1308 Cd (ALG)^2+^ (in the symmetric configuration),

−1718.0716 Cu (ALG)^2+^ < −1570.1307 Cd (ALG)^2+^(in the inverse configuration),

−1837.8551 (Cu-ALG-Cu)^4+^ < (Cd-ALG-Cd)^4+^ −1541.7832 (in the dimer configuration).

The bond lengths also exhibited a similar trend with greater IBSI of copper in the last configurations compared to cadmium. Hence, copper complexes generally display reduced bond lengths, more negative energy and high IBSI values, reflecting stronger interaction forces and superior stability in contrast to cadmium complexes. Consequently, the obtained findings are consistent with the results from the HOMO–LUMO analysis.^78^

In the third dimer arrangement, one carbonyl group binds to copper and another binds to cadmium. The bond length between the oxygen atom and copper is shorter than that with cadmium, and the O–Cu bond displays a higher IBSI value. This suggests that copper ion has stronger interaction forces and competes more efficiently than cadmium ion for the available biosorption sites on the proposed structure of alginate.

When comparing IBSI values across different complexes or systems, the relative difference in IBSI gives insight into the relative bond strength.^79,80^ A higher IBSI for metal–ligand bond implies that the metal is more strongly adsorbed compared to a system with a lower IBSI, which might suggest that the metal is weakly adsorbed and may be less stable in the complex. This indicates that complex (Cu-ALG-Cu)^4+^ has a stronger bond between the metal and the ligand, suggesting more effective biosorption or stronger binding. Conversely, the (Cd-ALG-Cd)^4+^ complex might have weaker biosorption, indicating that the metal ion is less tightly bound to the ligand.

Table S5† portrays the Mulliken charges of copper and cadmium both prior to and following their complexation with alginate in different configurations. The results demonstrate a significant decrease in the charge on each metal ion after complexation, indicating substantial electron transfer from alginate orbitals to the metal orbitals. A higher charge on cadmium after biosorption suggests a weak covalent interaction between the metal and the ligand. The metal remains strongly ionic, which reduces the possibility of forming a strong bond with the ligand. This may also indicate that the biosorption is primarily based on electrostatic forces rather than effective covalent bonds.

### 5.4. Binding energy

Table 6 summarizes the binding energies of Cu (ALG)^2+^, Cd (ALG)^2+^, (Cu-ALG-Cu)^4+^, (Cu-ALG-Cd)^4+^ and (Cd-ALG-Cd)^4+^. The calculated data reveals that an increase in binding energy is correlated with a greater absolute value of *E* complex, indicating a more powerful coordination and a stronger affinity between the adsorbate and adsorbent.^81,82^ Regarding the complexes' binding energies, the complex with the highest binding energy is (Cu-ALG-Cu)^4+^, followed by (Cu-ALG-Cd)^4+^, Cu (ALG)^2+^/inverse, Cu (ALG)^2+^/symmetry, Cd (ALG)^2+^/inverse, (Cd-ALG-Cd)^4+^, with Cd (ALG)^2+^/symmetry the lowest binding energy value. This result indicates that the proposed dimer as well as the monomer configurations of alginate have a stronger affinity for Cu^2+^ compared to Cd^2+^.

**Table 6:** Binding energies of Cu(ALG)^2+^/symmetry, Cu(ALG)^2+^/inverse, Cd(ALG)^2+^/symmetry, Cd(ALG)^2+^/inverse, (Cu-ALG-Cu)^4+^, (Cd-ALG-Cd)^4+^, (Cu-ALG-Cd)^4+^ complexes

Complex	*E*_total_ (Ha)	*E*_adsorbent_ (Ha)	*E*_adsorbat_ (Ha)	*E*_ads_ (kcal mol^−1^)
Cu (ALG)^2+^/symmetry	−1717.9626	−761.0647	−195.61	140.0178
Cu (ALG)^2+^/inverse	−1718.0716	−761.0647	−195.61	208.4207
Cd (ALG)^2+^/symmetry	−1570.1307	−761.0647	−47.83	107.4925
Cd (ALG)^2+^/inverse	−1570.1335	−761.0647	−47.83	108.6031
(Cu-ALG-Cu)^4+^	−1837.8551	−1445.9495	−195.61	430.2512
(Cd-ALG-Cd)^4+^	−1541.7832	−1445.9495	−47.83	107.7598
(Cu-ALG-Cd)^4+^	−1689.8148	−1445.9495	−243.441	266.2865

## Conclusion

This study proposed and optimized cadmium and copper ions elimination from aqueous solutions using a brown alga; *Cystoseira sedoide*, without any treatment. Batch biosorption assays revealed that Cd^2+^ and Cu^2+^ biosorption onto *C. Sedoide* was independent of solution pH, biosorption dose and initial metal ion concentration.

Freundlich model was adequately used to characterize the heavy metals biosorption. The achieved adsorption capacities were 23.78 and 14.66 mg g^−1^ for Cd^2+^ and Cu^2+^, respectively. *Cystoseira sedoide* performance exceeded that of the commercial activated carbon. The biosorption of the targeted heavy metals was spontaneous and followed a pseudo-second order kinetics. Furthermore, the theoretical investigation using DFT computations was performed with the assumption that organometallic complexes were formed between the heavy metals and two structures reflecting alginate monomer and dimer, as well. This computational method was undertaken to investigate the biosorption difference between the two metal ions. Based on the electrostatic potential maps, the oxygen atom of the carbonyl groups were the main electrophilic attack centers in the proposed structures. Results revealed that the bond lengths between the heavy metals and the alginate optimized structures decreased from cadmium to copper complexes. The energy gap was lower for Cu complexes than for Cd complexes, depicting a strong interaction for copper. These results were further supported by the high IBSI values for copper complexes. Theoretical findings revealed that the stability and interaction properties of the complexes are modulated by the metal type and the specific configuration of the complexes, suggesting the exploration of more extended polysaccharide structure to provide an accurate explanation of the excellent experimental results. This is the objective of an-ongoing research. This study open up significant new opportunities for algal biomass as a rapid, efficient and low-cost biosorbent, especially for the countries in the Mediterranean basin. This naturally abundant alga might be considered as a valuable material for water decontamination. Besides the revealed benefits of using *Cystoseira sedoide* for the removal of heavy metals, the biomass can be reused as a feedstock for the production of biochar or bioethanol, leading to economic and environmental sustainability when implemented on a large scale.

## Data availability

The data supporting this article have been included as part of the ESI.†

## Conflicts of interest

There are no conflicts of interest to declare.

## Supplementary Material

RA-014-D4RA07331B-s001
