# Supplementary material for: Exploring the efficacy of Cystoseira sedoide alga for cadmium and copper biosorption: an integrated experimental and computational study
Source: RSC Adv. 2024 Dec 10;14(52):38721–38. doi: 10.1039/d4ra07331b (PMC11629872; doi:10.1039/d4ra07331b)
Supplement: RA-014-D4RA07331B-s001 [file RA-014-D4RA07331B-s001.pdf]

Supplementary Information (SI) for RSC Advances.
This journal is © The Royal Society of Chemistry 2024

# Exploring the Efficacy of *Cystoseira sedoide* alga for Cadmium and Copper Biosorption: An Integrated Experimental and Computational Study

Nadjette BENGOURNA[a], Karima BELGUIDOUM[b], Dounya KHALLA[c], Mouna NACEF[c], Imane KOUADRI[c], Aida BENHAMIDA[a], Habiba AMIRA-GUEBAILIA[b], Alaa eddine BROUK[c], Abed Mohamed AFFOUNE[c], Hamid SATHA[a]

[a] Laboratoire des Silicates, Polymères et des Nanocomposites (LSPN), Université 8 Mai 1945, Guelma, BP 401, Guelma, 24000, Algeria.

[b] Laboratoire de Chimie Appliquée (L.C.A), Université 8 Mai 1945, BP 401, 24000 Guelma, Algeria

[c] Laboratoire d'Analyses Industrielles et Génie des Matériaux (L.A.I.G.M), Université 8 Mai 1945 Guelma, BP 401 Guelma 24000, Algeria.

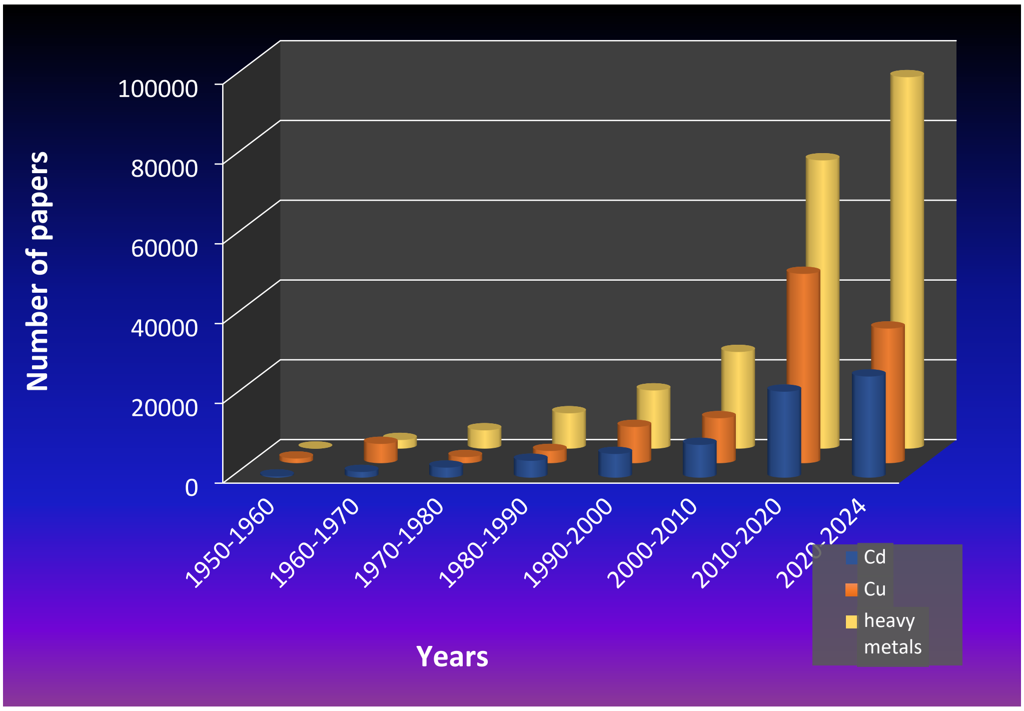


**Figure S1.**Published documents per year related to heavy metals adsorption (from Science Direct database). Keywords: Heavy metals, Cadmium, Copper, Adsorption.

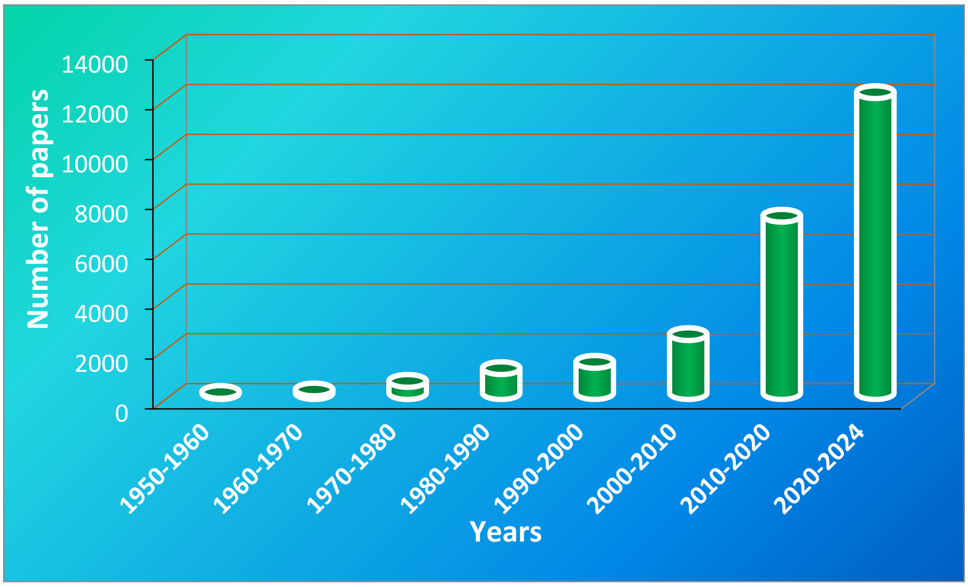


**Figure S2.**Published documents per year related to the use of algae in the adsorption (from Science Direct database). Keywords: Algae, Adsorption.

**Table S1.**Functional groups of *Cystoseira sedoide* alga before and after $Cd^{2+}$ and $Cu^{2+}$biosorption.

| Wavelength ($cm^{-1}$) | | | Functional groups |
|---|---|---|---|
| *C. sedoide* | Cu /*C. sedoide* | Cd/ *C. sedoide* | |
| 868.34 | 866.48 | 864.62 | C-H out of plane bending vibrations in aromatic groups |
| 1011.77 | 1009.90 | 1011.77 | C-O bonds of saccharide structure |
| 1410.37 | 1410.37 | 1408.51 | -COO- symmetric stretching vibrations |
| 1596.63 | 1585.46 | 1596.63 | -COO- asymmetric stretching vibrations |
| 2919.10 | ------ | ------ | -CH stretching vibrations (υCH, alkyl). |
| 3265 | 3265 | 3265 | -OH stretching vibration |

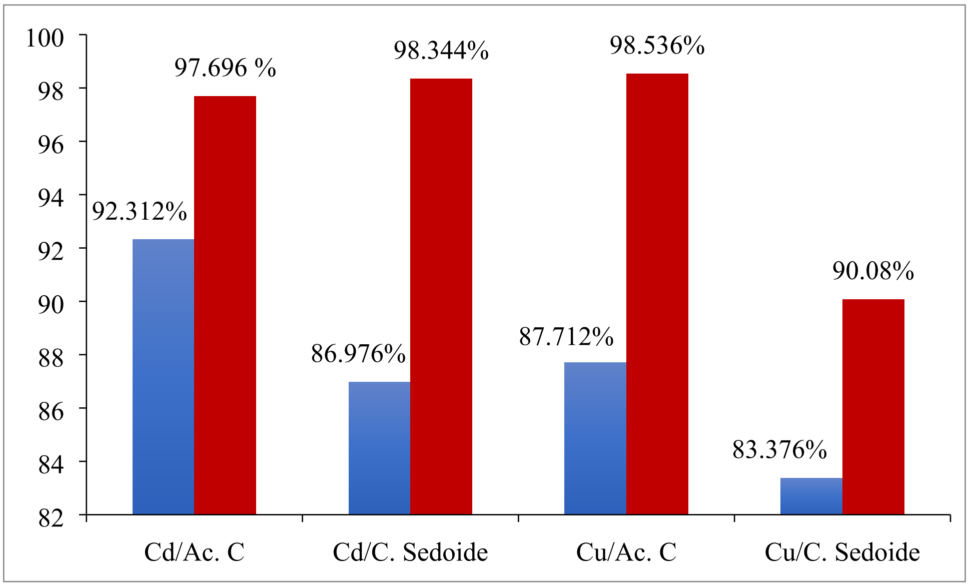


**Figure S3.** Effect of the solution stirring on the adsorption of $Cd^{+2}$ and $Cu^{+2}$ on *C. sedoide* and Ac.C. Blue: without stirring and red: with stirring at 300 rpm.

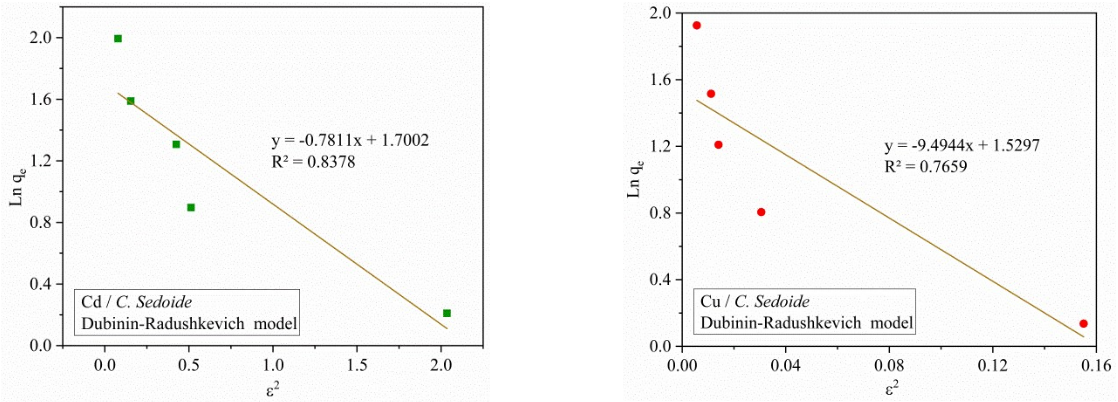


**Figure S4.** Linearized plots of Dubinin-Radushkevich (D-R) model for different isotherm models for $Cd^{2+}$ ions biosorption on *C. sedoide*

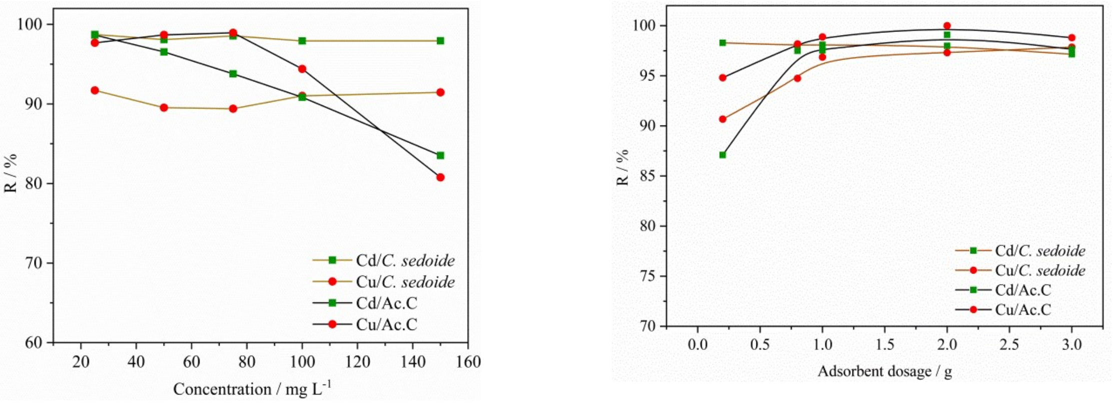

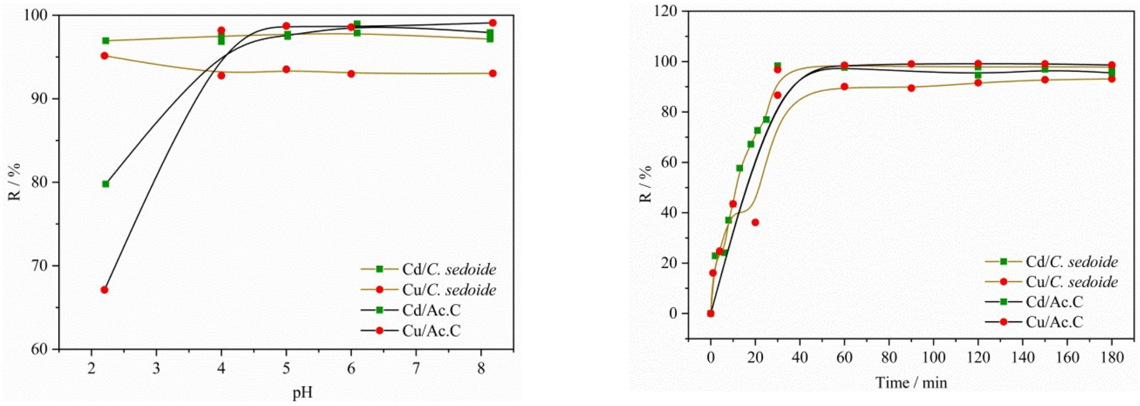


**Figure S5.** Effect of initial concentration, *C. sedoide* alga and Ac.C doses, pH, and temperature on the removal rate of $Cd^{2+}$ and $Cu^{2+}$ ions

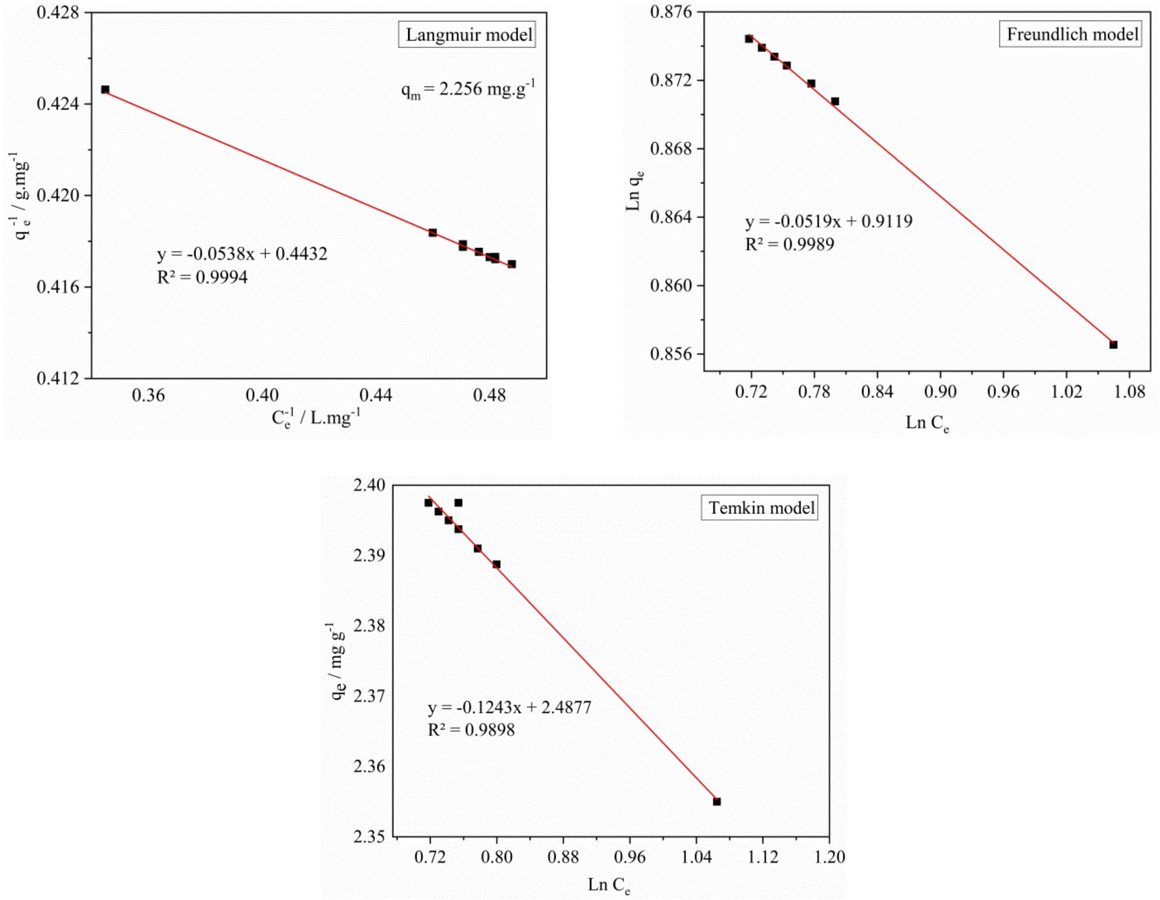


**Figure S6**. Linearized plots of different isotherm models for $Cd^{2+}$ ionsbiosorptionon *C. sedoise* in the presence of $Cu^{2+}$ ions.

**Table S2.**Summary of Dubinin-Radushkevich (D-R) model used for the adsorption of $Cd^{2+}$ and $Cu^{2+}$.

| Dubinin-Radushkevich equation | $q_e = q_{DR}\, exp\big((-B_{DR}\, \varepsilon_{DR}^{2}\big)$ |
|---|---|
| Dubinin-Radushkevich linearized equation | $\ln q_e = \ln q_{DR}\, \beta\varepsilon^2$ |
| Polanyi potential | $\varepsilon_{DR} = RT\ln\left(\frac{C_s}{C_e}\right)$ |
| The mean free energy of biosorption(E) | $E = \frac{1}{\sqrt{2\,\beta_{DR}}}$ |
| E significance | 8 kJ/mol<E<16 kJ/mol→Chemisorption |
| | E<8 kJ/mol→Physisorption |
| Results | $Cd^{2+}$/ *C. Sedoide*: E= 14.2 kJ/mol |
| | $Cu^{2+}$/ *C. Sedoide*: E= 8.45 kJ/mol |
| | $Cd^{2+}$/ Ac.C : E= 11.2 kJ/mol |
| | $Cu^{2+}$/ Ac.C : E= 15.58 kJ/mol |

**Table S3.** Thermodynamic parameters of $Cd^{2+}$ and $Cu^{2+}$biosorption on *C. Sedoide*

| Thermodynamic parameters | | Cadmium | Copper |
|---|---|---|---|
| $\Delta H^0$ (kJ.mol$^{-1}$) | | 3.0899 | 9.36 |
| $\Delta S^0$ (kJ.mol$^{-1}$.K$^{-1}$) | | 0.042 | 0.050 |
| | 293 K | - 9.234 | -5.227 |
| | 303 K | - 9.674 | -5.931 |
| $\Delta G^0$(kJ.mol$^{-1}$) | 313 K | -10.106 | -6.046 |
| | 323 K | -10.540 | -6.559 |
| | 333 K | -10.902 | -7.427 |

**Figure S7.** Linearized plots of different isotherm models for $Cd^{2+}$ ions biosorption on *C. sedoise* in the presence of $Cu^{2+}$ ions

**Table S4.** Competitive biosorption parameters of $Cd^{2+}$ on *C. sedoide* in the binary-metal system

| | | Biosorption capacity ratio | Biosorption reduction rate |
|---|---|---|---|
| Metal | Adsorption systems | $q_{ratio} = \frac{q_{m,i}^{mix}}{q_{m,i}^{0}}$ | $\Delta Y = \frac{q_{m,i}^{0} - q_{m,i}^{mix}}{q_{m,i}^{0}} X 100\ \%$ |
| $Cd^{2+}$ | $Cd^{2+}$, $Cu^{2+}$ | 0.208 | 79.25 |

**Table S5.** The Mulliken charge of $Cd^{2+}$ and $Cu^{2+}$ before and after complexation with alginate in different configurations.

| **Metal** | **Charge** | |
|---|---|---|
| | **Before complexation** | **After complexation** |
| $Cu(ALG)^{+2}$/Symetrie | 2 | 0.969 |
| $Cu(ALG)^{+2}$/Inverse | 2 | 1.096 |
| $Cd(ALG)^{+2}$/Symetrie | 2 | 1.682 |
| $Cd(ALG)^{+2}$/Inverse | 2 | 1.676 |
| $(Cu\text{-}ALG\text{-}Cu)^{+4}$ | 4 | $Cu_{45}$ :0.809<br>$Cu_{44}$: 0.815 |
| $(Cd\text{-}ALG\text{-}Cd)^{+4}$ | 4 | $Cd_{45}$:1.871<br>$Cd_{44}$: 1.849 |
| $(Cu\text{-}ALG\text{-}Cd)^{+4}$ | 4 | $Cd_{44}$:1.868<br>$Cu_{45}$: 0.802 |
